# A powerful and versatile new fixation protocol for immunostaining and in situ hybridization that preserves delicate tissues

**DOI:** 10.1186/s12915-024-02052-3

**Published:** 2024-11-04

**Authors:** Carlos Guerrero-Hernández, Viraj Doddihal, Frederick G. Mann, Alejandro Sánchez Alvarado

**Affiliations:** 1 Stowers Institute for Medical Research, Kansas City, MO USA; 2 Howard Hughes Medical Institute, Kansas City, MO USA

**Keywords:** WISH/FISH, In situ hybridization, Immunostaining, Regeneration, Wound healing, Blastema, Immunofluorescence, Planaria, Killifish

## Abstract

**Background:**

Understanding how genes function to heal wounds and restore lost tissue is essential for studying regeneration. Whole-mount in situ hybridization (WISH) is a powerful and widely used technique to visualize the expression patterns of genes in different biological systems. Yet, existing methods to permeabilize samples for WISH can damage or destroy fragile regenerating tissues, thereby preventing such experiments.

**Results:**

Here, we describe a new protocol for in situ hybridization (ISH) and immunostaining in the highly regenerative planarian *Schmidtea mediterranea*. This new Nitric Acid/Formic Acid (NAFA) protocol is compatible with both the assays and prevents degradation of the epidermis and regeneration blastema. The NAFA protocol achieves this without the use of proteinase K digestion which likely leads to better preservation of antigen epitopes. We show that the NAFA protocol successfully permits development of chromogenic and fluorescent signals in situ, while preserving the anatomy of the animal. Furthermore, the immunostaining of different proteins was compatible with the NAFA protocol following fluorescent in situ hybridization. Additionally, the tissue fixation protocol was easily adapted for regenerating killifish tail fin, which yielded better ISH signal with minimal background.

**Conclusions:**

Thus, the NAFA protocol robustly preserves the delicate wounded tissues while also facilitating probe and antibody penetration into internal tissues. Furthermore, the fixation protocol is compatible for WISH on regenerating teleost fins suggesting that it will be a valuable technique for studying the processes of wounding response and regeneration in multiple species.

**Supplementary Information:**

The online version contains supplementary material available at 10.1186/s12915-024-02052-3.

## Background

Regeneration is the ability to restore tissues or organs lost to injury and it varies widely among metazoans. While some animals like fish and axolotls are capable of regenerating certain appendages and tissues, others like planarian flatworms and *Hydra* are capable of whole-body regeneration. [1–4]. The cellular and molecular activities that drive regeneration are not yet fully understood. Understanding the molecular changes that take place in the delicate wound epidermis and newly produced tissue is essential to revealing the molecular basis of regeneration.

RNA in situ hybridization (ISH) is a key method for studying gene expression patterns both during homeostasis and regeneration [5, 6]. Unlike bulk and single-cell RNA-sequencing methods, ISH provides extensive detail by visualizing gene expression patterns in their native tissue contexts [5, 7]. Furthermore, because this method does not require transgene expression, it can be performed on wildtype research organisms that do not yet have developed genetic toolkits. As such, it is particularly useful for research questions being pursued in diverse research organisms [8].

The freshwater planarian *S. mediterranea* can regrow a complete animal from a body fragment that is less than 1% of its original size [3]. This remarkable capacity for regeneration has attracted the attention of generations of biologists. Its study has required the development of methods to detect, measure, and visualize the cells and molecules underpinning regeneration. ISH has been a primary tool for studying the biology of planarian stem cells and regeneration [9, 10]. Yet, current ISH protocols have several shortcomings. Penetration of probes into tissue for whole-mount in situ hybridization (WISH) is difficult to achieve. As such, permeability is increased through tissue digestion with proteinase K and through aggressive treatment with the mucolytic agent N-acetyl cysteine (NAC) [9, 10]. These harsh treatments can damage or destroy delicate tissues and often result in the shredding of both the epidermis and the regeneration blastema (the fragile unpigmented tissue at the wound edge which gives rise to lost body parts). Moreover, immunological assays could be weak on samples prepared by this protocol, likely because proteinase digestion disrupts target epitopes. Other protocols have been developed for fixing whole planarians that preserve the gross anatomical structures and perform well in immunological assays, but those methods are not compatible with ISH [11]. An ideal method would preserve delicate tissues and permit the simultaneous analysis of RNA and protein expression patterns.

Here, we present a new fixation protocol for ISH and immunofluorescence in planarians. We have combined approaches from several fixation techniques into a Nitric Acid/Formic Acid (NAFA) strategy for sample preparation that better preserves the delicate epidermis and blastema than previous methods do [11–13]. This NAFA protocol does not include a protease digestion, providing increased compatibility with immunological assays, while not compromising ISH signal. We also show this protocol can be easily adapted for ISH studies in the regenerating killifish tail fin. Thus, the protocol is potentially applicable to a wide range of species and particularly facilitates the study of delicate tissues via ISH and immunofluorescence.

## Results

**Fig. 1:**
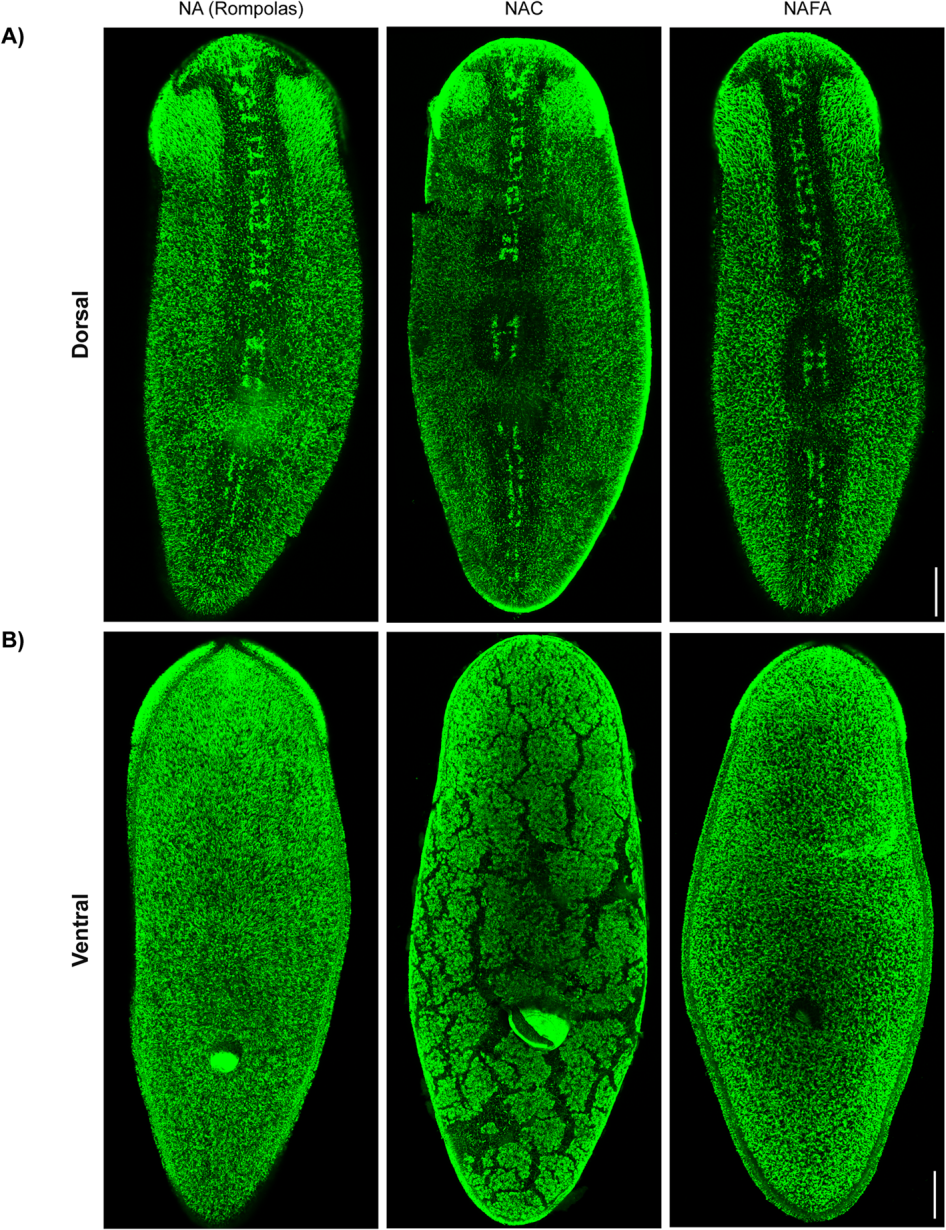
Epidermal integrity is preserved with Nitric Acid/Formic Acid (NAFA) protocol. Images are immunofluorescence of cilia stained with anti-acetylated tubulin antibody on the **A** dorsal and **B** ventral surfaces. For all images, animals are oriented with anterior at the top. Maximum intensity projection of confocal image stacks, scale bars: 100 μm

We sought to create a new fixation protocol for planarians that would be compatible with both ISH and antibody-based assays while preserving the structural integrity of the animals. We reasoned that combining the acid treatment strategies of a variety of protocols could make the samples compatible with multiple applications [9, 11, 12, 14]. We also included the calcium chelator ethylene glycol-bis(β-aminoethyl ether)-N,N,N′,N′-tetraacetic acid (EGTA) to inhibit nucleases and preserve RNA integrity during sample preparation [15]. To determine the extent to which the new combination of acids preserved the samples, we used the integrity of the epidermis as a proxy for tissue preservation, and we visualized it immunostaining cilia with an anti-acetylated tubulin antibody [16]. We tested a *N*itric *A*cid/*F*ormic *A*cid (NAFA) fixation and compared it against two well established fixation protocols in the field, NA (Rompolas) [11] and N-Acetyl-Cysteine (NAC) [9]. We found that the integrity of the epidermis is well preserved in both the NA (Rompolas) and NAFA protocols, whereas noticeable breaches of integrity were detected when the protocol using the mucolytic compound NAC was tested (Fig. 1). We concluded from these results that the NAFA protocol worked as well as the NA (Rompolas) protocol and preserved the sample considerably better than the NAC protocol did.

**Fig. 2:**
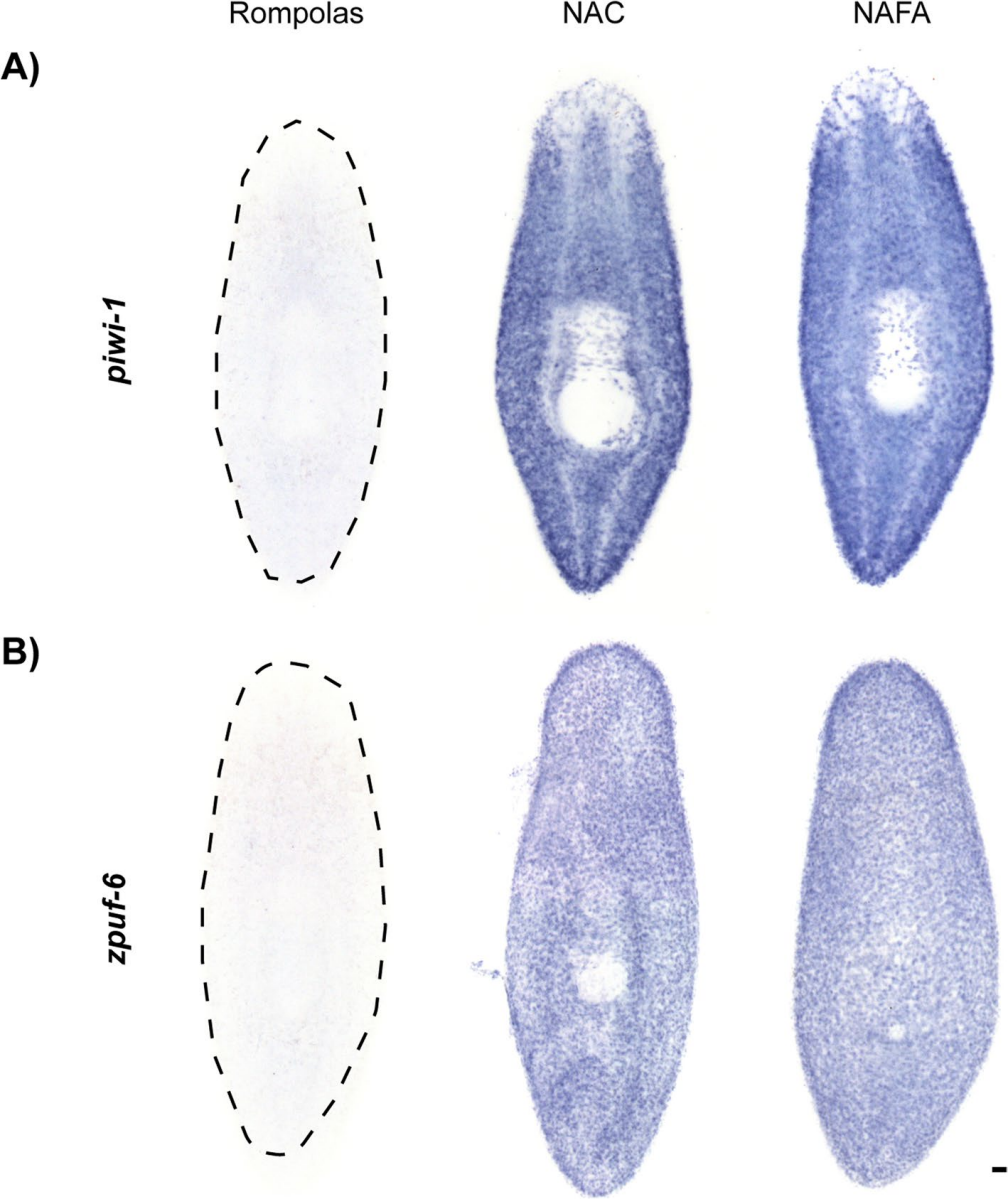
The NAFA protocol is compatible with chromogenic in situ hybridization. Chromogenic in situ hybridizations were developed with NBT/BCIP and brightfield images were collected on a stereomicroscope. **A ***piwi-1* (neoblasts) and **B ***zpuf-6* (epidermal progenitors). Animals are oriented with anterior at the top. Scale bars: 100 μm

Given the success of the anti-acetylated tubulin antibody staining, we tested whether the NAFA protocol could be used for ISH assays. To ensure the NAFA protocol allows antisense RNA probe penetration into tissues, we chose genes known to mark the internal neoblast cell population (*piwi-1*), and a more external cell population, a subset of the epidermal progenitors (*zpuf-6*) [17, 18]. First, we tested whether the expression of *piwi-1* and *zpuf-6* could be detected via chromogenic WISH (Fig. 2). While the NAFA and NAC protocols produced indistinguishable patterns of expression for the two genes, we could not observe any *piwi-1* and *zpuf-6* signal with the NA (Rompolas) protocol (Fig. 2A, B). These experiments also revealed epidermal damage when NAC was used (Fig. 2B). To further investigate epidermal integrity and WISH signal, we performed chromogenic WISH for *zpuf-6* using the NAC and NAFA protocols (Additional file 1: Fig. S1) then sectioned the animals afterwards for histological analysis. The sections revealed that the outermost layer with *zpuf-6* + cells was intact when using the NAFA protocol but damaged by the NAC protocol (Fig. S1A and S1B). Also, we tested whether three different carboxylic acids (formic acid, acetic acid, and lactic acid) can be used in the NAFA protocol. We performed chromogenic WISH for *piwi-1*, *zpuf-6*, in addition to markers of the central nervous system (*pc2*) [19, 20], and gastrovascular system (*porcupine*) [21]. All showed similar expression patterns in both the NAFA and NAC protocols (Additional file 2: Fig. S2). While all three carboxylic acids can be used to determine gene expression patterns and are effective across multiple transcripts, we chose to use formic acid because it has the simplest chemical structure. We conclude from these findings that the new NAFA protocol both preserves epidermis integrity and can be used to detect gene expression in different planarian tissues via WISH.

**Fig. 3:**
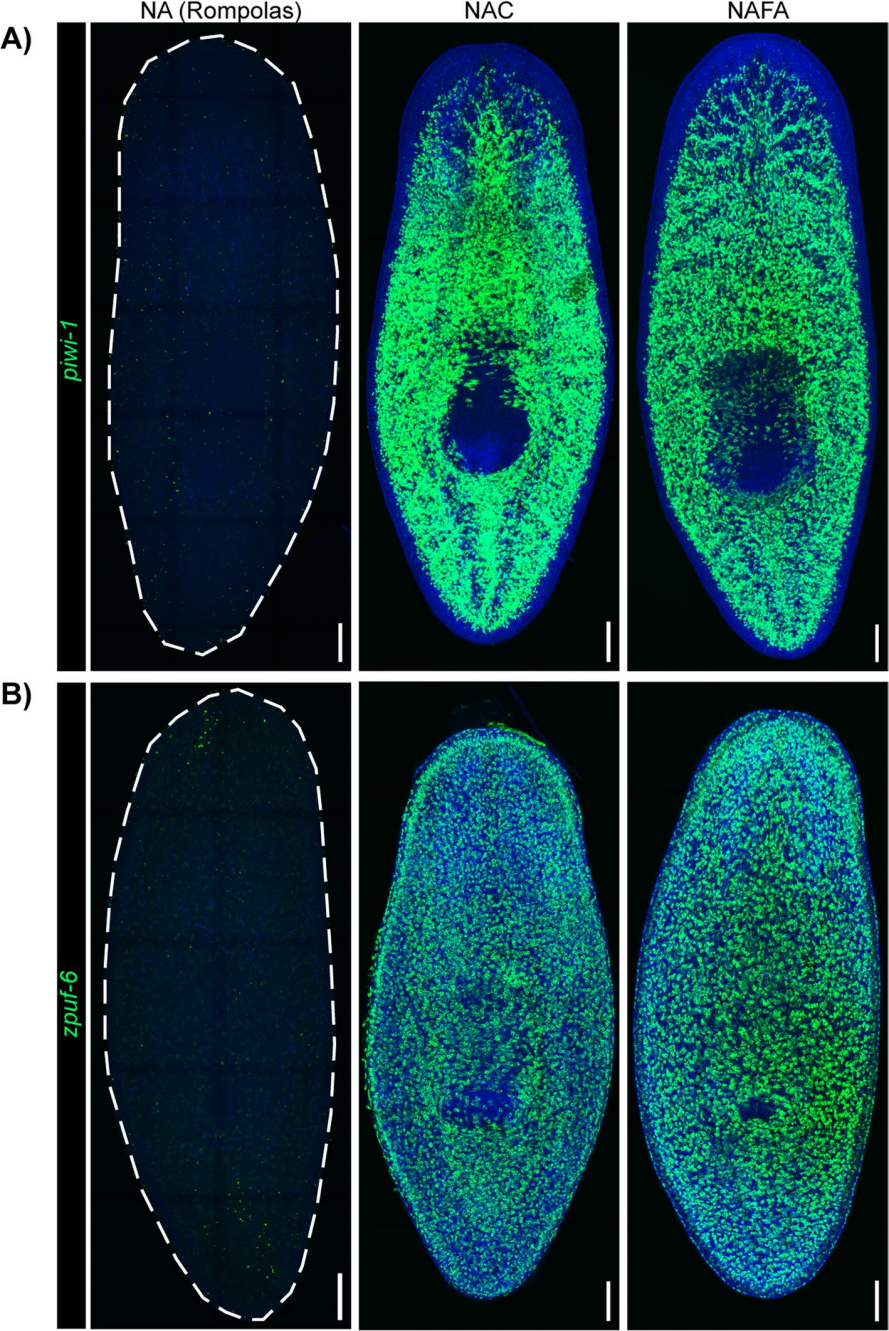
NAFA is compatible with fluorescent in situ hybridization. FISH (green) of **A ***piwi-1* (neoblasts) and **B ***zpuf-6* (epidermal progenitors). Animals were co-stained with DAPI (blue). Maximum intensity projection of confocal images, scale bars: 100 μm

**Fig. 4:**
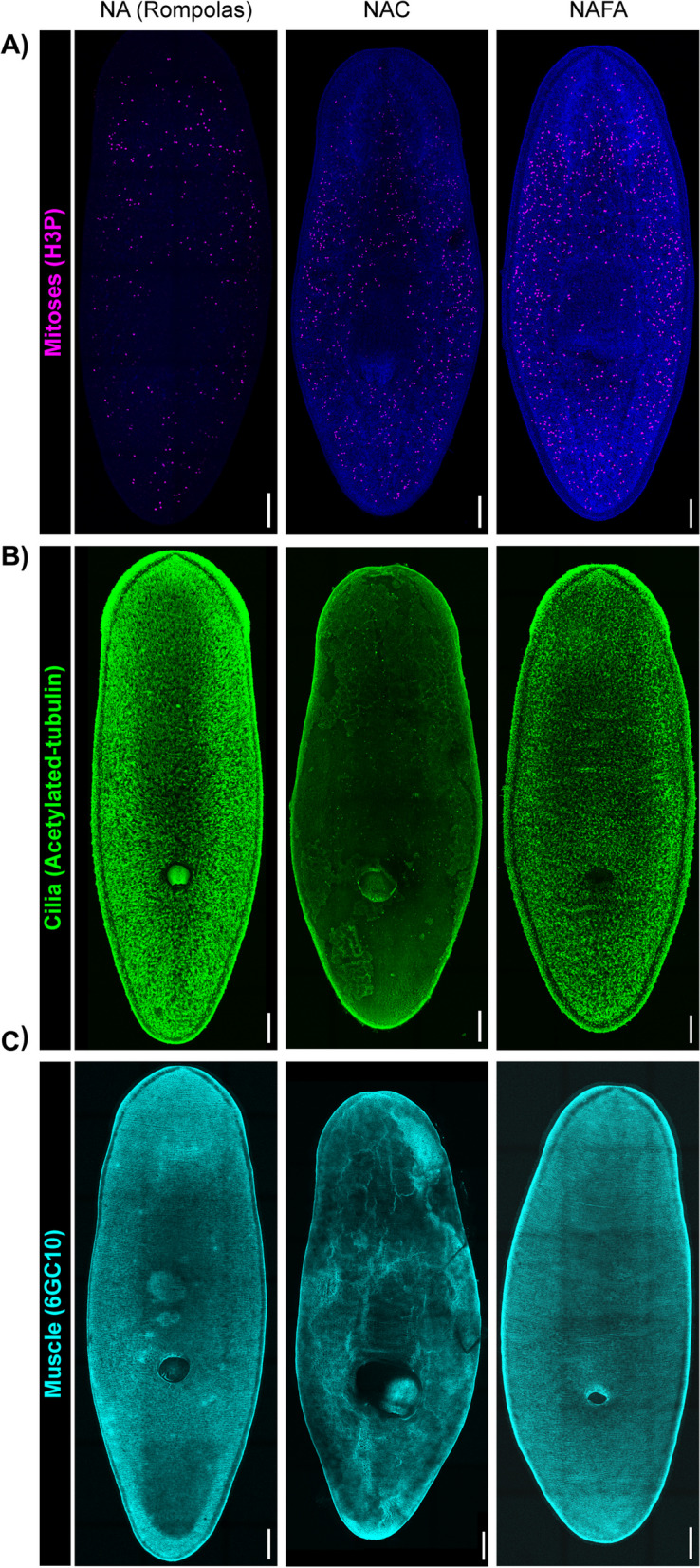
NAFA protocol enables immunofluorescent detection after fluorescent in situ. Immunostaining of **A** mitoses by anti-phosphorylated Histone H3 (magenta) co-stained with DAPI (blue), **B** cilia by anti-acetylated tubulin (green), and **C** muscle by 6G10-2C7 (cyan). Maximum intensity projection of confocal images, scale bars: 100 μm

Next, we investigated whether we could use the new NAFA protocol in planaria to carry out fluorescent in situ hybridization (FISH) in tandem with immunostaining. Using confocal microscopy, we detected the neoblast and epidermal progenitor markers *piwi-1* and *zpuf-6*, respectively (Fig. 3). The intensity of the *piwi-1* fluorescent signal was indistinguishable between the NAC and NAFA protocols but much weaker for the NA (Rompolas) protocol (Additional file 3: Fig. S3). Furthermore, confocal microscopy showed that the epidermis was damaged with the NAC protocol but was not visibly affected when using the NAFA protocol (Fig. 3B). After whole-mount FISH, we immunostained for mitotic cells with an antibody that recognizes the Serine-10 phosphorylated form of histone H3 (anti-H3P) [22, 23]. While we did not observe statistically significant differences in H3P density among the protocols (Additional file 4: Fig. S4A), the anti-H3P antibody showed brighter signal with the NAFA protocol when compared to both Rompolas and NAC protocols (Fig. 4A, Additional file 4: S4B, S4C). Therefore, NAFA is highly compatible with tandem FISH and immunostaining.

Next, we sought to more thoroughly characterize the ability of the three protocols to label external and internal tissues by immunofluorescence using antibodies against acetylated tubulin and Smed-6G10 [24]. As in prior experiments, the NA (Rompolas) and NAFA protocol preserved the cilia while they were damaged in the NAC protocol (Fig. 4B). In case of the muscle antibody, we observed that all the three protocols produced qualitatively similar staining pattern (Fig. 4C). However, NAC treatment sometimes damaged the body wall musculature resulting in inconsistent stainings when compared to the NAFA protocol (Additional file 5: Fig. S5). The NAFA protocol retained tightly packed evenly spaced muscle fibers, outermost circular muscle fibers, while NAC treatment disrupted the integrity of the muscle fibers and at places lost the circular fibers (Additional file 6: Fig. S6A). To further compare the muscle staining between the NAC and NAFA protocols, we imaged the internal gut musculature and observed that the NAC protocol produced crisper stainings compared to the NAFA protocol (Additional file 6: Fig. S6B). Similarly, we evaluated both protocols’ compatibility with staining protonephridia, another internal structure which is also labeled by the anti-acetylated tubulin antibody. This approach allowed us to compare external vs. internal staining using the same antibody. We observed similar staining of protonephridia in both the protocols, but the epidermal cilia were damaged in the NAC protocol while the NAFA protocol preserved the cilia (Additional file 6: Fig. S6C and S6D). Thus, the NAFA protocol is well suited to studying fragile external structures and most internal structures.

**Fig. 5:**
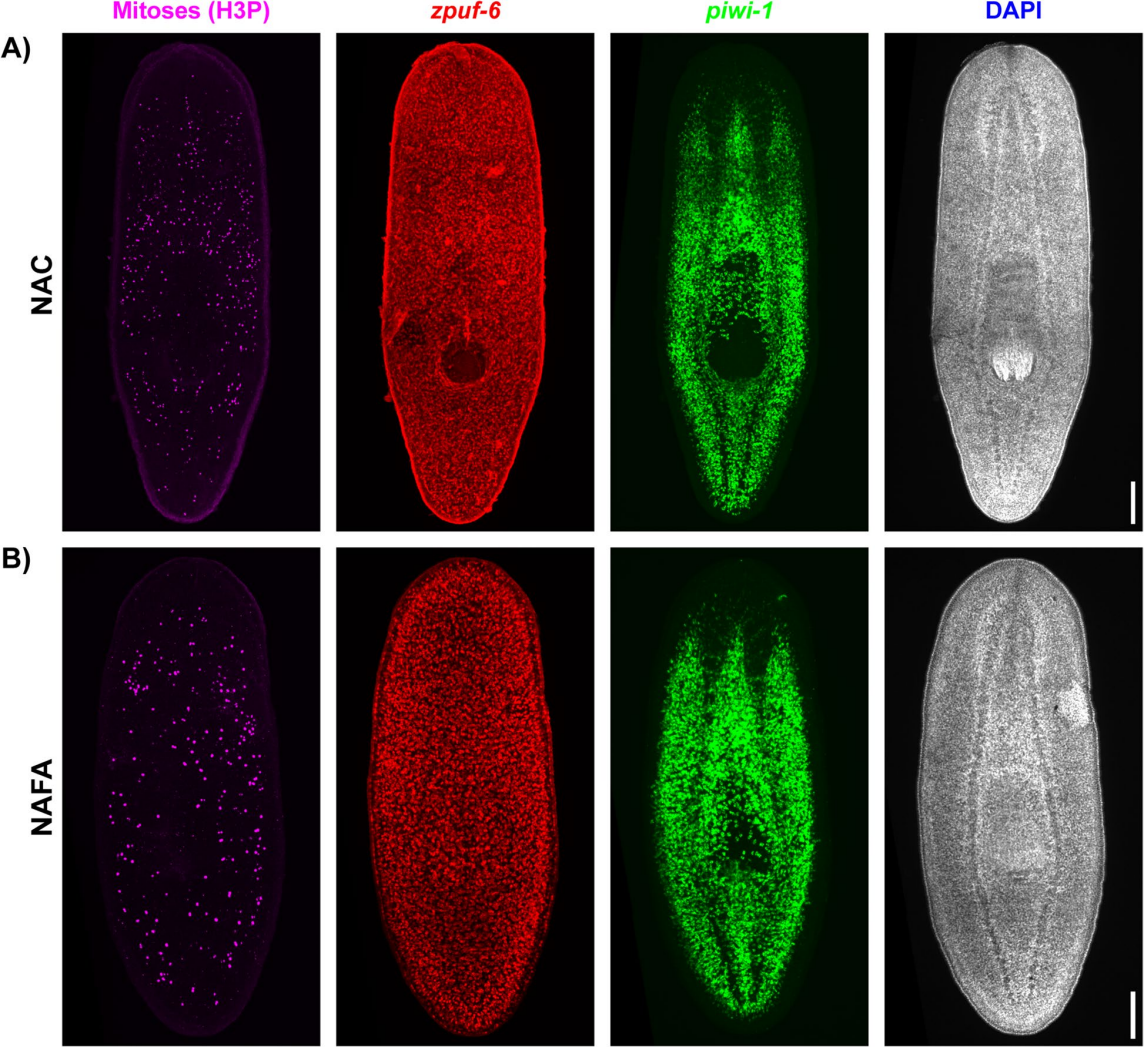
NAFA protocol is compatible with double fluorescent in situ hybridization followed by immunofluorescence. **A**, **B** FISH of *zpuf-6* (red) and *piwi-1* (green). Immunostaining of mitoses by anti-phosphorylated Histone H3 (magenta). Animals were co-stained with DAPI (gray). Maximum intensity projection of confocal image stacks, scale bars: 100 μm

We then assessed if we could use the new NAFA protocol to develop two-color FISH with two different RNA probes using *piwi-1* and *zpuf-6*. Because the NA (Rompolas) protocol is not compatible with ISH, we only compared the NAFA and NAC protocols to each other. First, we detected *zpuf-6* gene expression followed by *piwi-1*. We used confocal microscopy to image the samples and observed similar expression patterns of *piwi-1* in both protocols. However, the NAFA protocol showed a clearer expression pattern of the epidermal progenitor *zpuf-6* likely because the integrity of the epidermis was preserved (Fig. 5A, B). After the double FISH, we explored the mitotic cells in the same samples using anti-H3P antibody. We observed comparable densities of H3P nuclei for both the protocols (Fig. 5A, B, and Additional file 7: Fig. S7). Therefore, NAFA is compatible with two-color FISH and immunostaining.

**Fig. 6:**
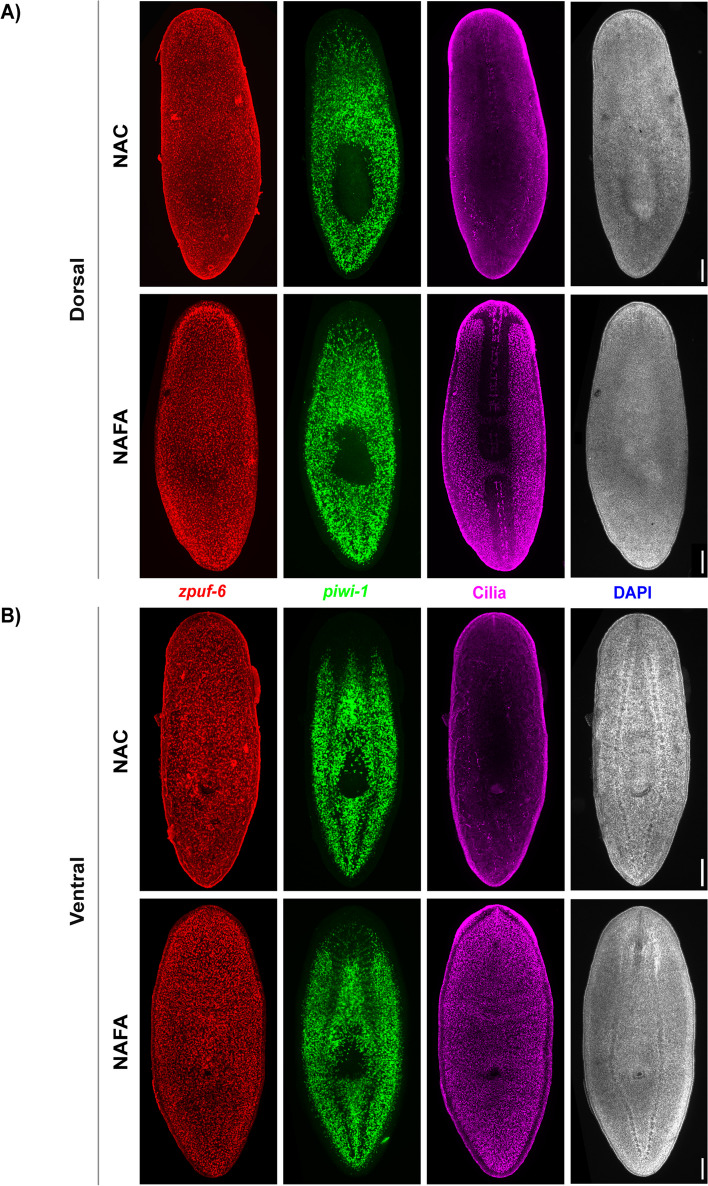
NAFA protocol maintains cilia on the epidermis after double fluorescent in situ hybridization. **A**, **B** FISH of *zpuf-6* (red) and *piwi-1* (green) and immunostaining of cilia by anti-acetylated tubulin (magenta). Animals were co-stained with DAPI (gray). **A** Dorsal view. **B** Ventral view. Maximum intensity projection of confocal image stacks, scale bars: 100 μm

To confirm that the NAFA protocol preserves the epidermis even after double FISH of *piwi-1* and *zpuf-6*, we subsequently performed immunostaining for cilia. The confocal images of the dorsal and ventral sides of planarians after two-color FISH showed well preserved cilia with the NAFA protocol. In contrast, we failed to detect the same pattern of cilia in planarians treated with NAC protocol (Fig. 6A, B). Hence, the NAFA protocol not only preserves the internal structures akin to the NAC protocol but also maintains epidermal integrity even after the strenuous protocol of labeling two separate transcripts and a protein.

**Fig. 7:**
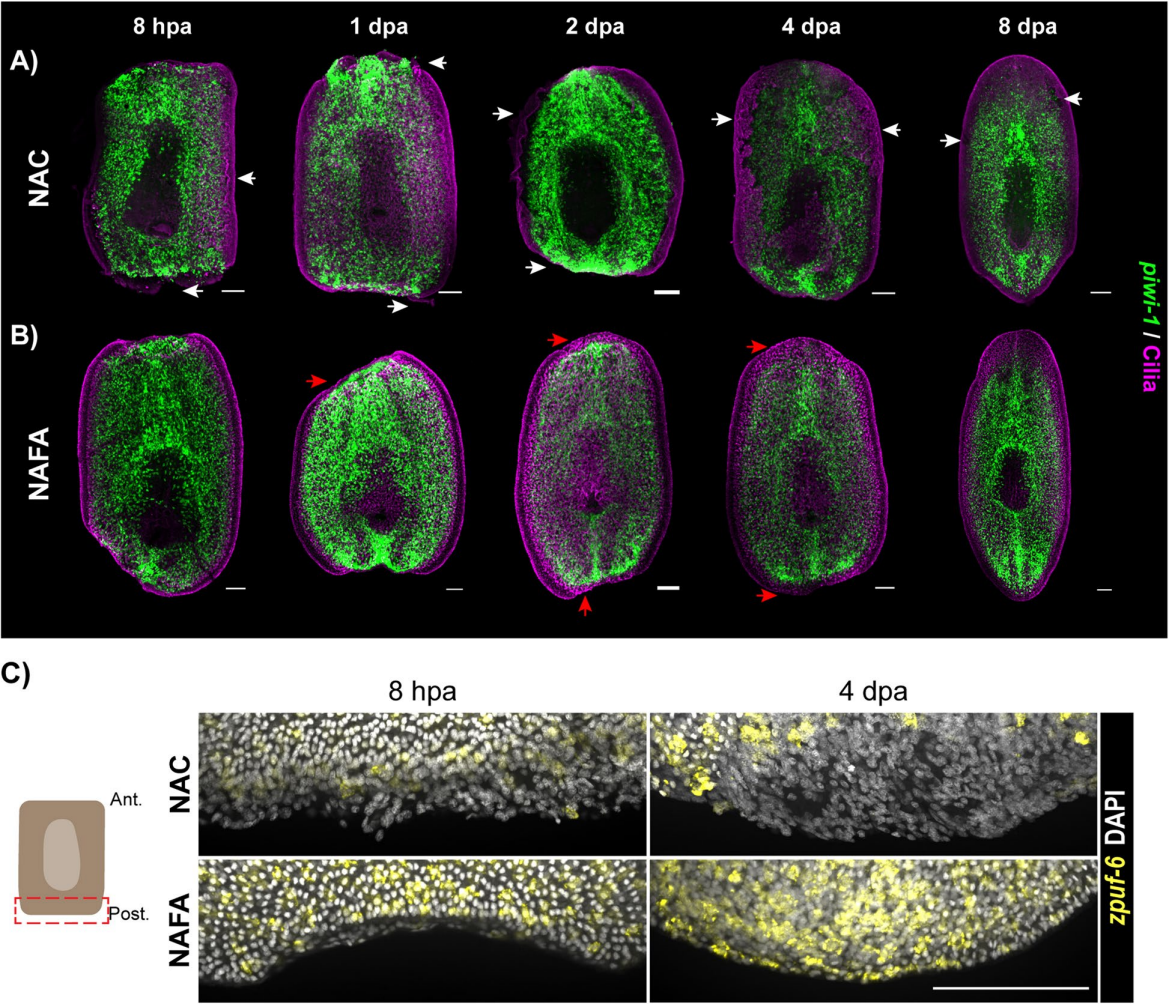
NAFA protocol maintains epidermal integrity during regeneration. **A**, **B** FISH of *piwi-1* (green) and immunostaining of cilia by anti-acetylated tubulin (magenta). **A** White arrows indicate damage to the epidermis and blastema for animals processed with the NAC protocol. **B** Red arrows at the blastema show epidermal integrity during regeneration. **C** FISH of *zpuf-6* (yellow) and DNA staining with DAPI (white). Maximum intensity projection of confocal images, scale bars: 100 μm

We next tested if we could use the NAFA protocol to study the wounding response during planarian regeneration without damaging the fragile epidermis or nascent blastema tissues. We performed FISH of *piwi-1* and the immunostaining of cilia on trunk fragments 8 h post amputation (hpa) and at 1, 2, 4, and 8 days post amputation (dpa) to assay for epidermal integrity (Fig. 7A, B). Confocal images showed that epidermal integrity was compromised by the NAC protocol, while the NAFA samples had very clear staining of cilia on trunks throughout regeneration (Fig. 7A, B). Remarkably, while the *piwi-1* FISH pattern was similar between the NAFA and NAC protocols, the NAFA-fixed fragments exhibited an area of undifferentiated tissue that could not be detected in the NAC fragments (compare white arrows in Fig. 7A to red arrows in Fig. 7B). We next imaged the blastema at higher magnification with confocal microscopy. These images reinforced that the NAFA protocol preserves the wound epidermis and the blastema, while it was heavily damaged by the NAC protocol (Fig. 7C and Additional file 8: Fig. S8).

To independently verify preservation of the wound epidermis when using the NAFA protocol, we carried out Acid Fuchsin Orange G staining (AFOG). Cryosections of animals fixed with the NAC protocol showed extensive damage to the epidermis, while the NAFA-treated samples had well organized epidermis with tall cells and distinct basal lamina (red arrows) (Additional file 9: Fig. S9A and Fig. S9B). The wound epidermis (8 hpa) was damaged and at times lost in NAC-treated sections but was retained in NAFA-treated sections (Additional file 9: Fig. S9A). Similarly, the blastema at 4 dpa was better preserved upon NAFA treatment (Additional file 9: Fig. S9B). Taken together, the data show that the NAFA protocol is well suited to study wounding responses and blastema formation during regeneration.

**Fig. 8:**
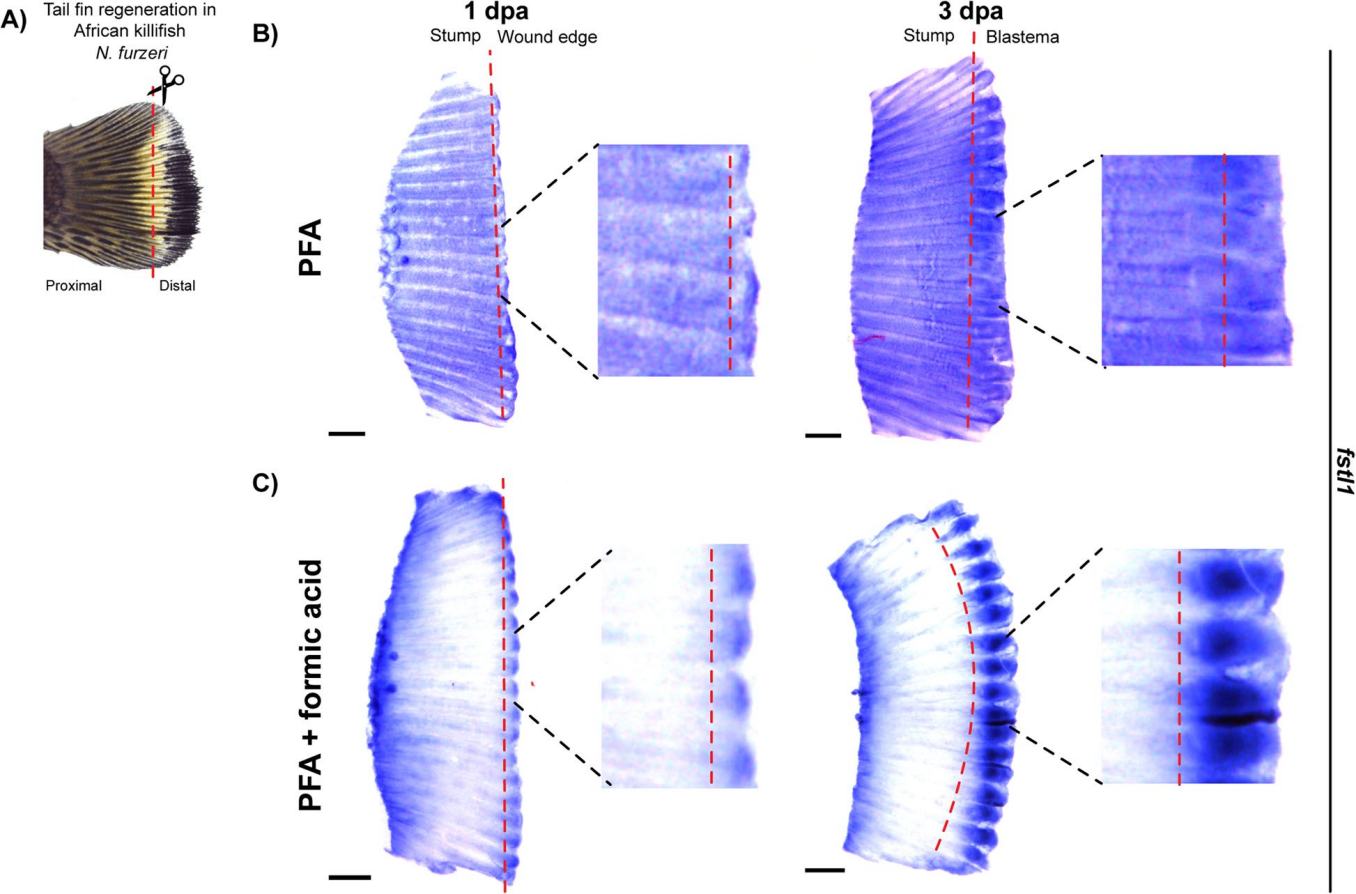
ISH in killifish tail fin yields better signal to noise ratio when fixed with PFA + formic acid. **A** Morphology of the tail fin in the African killifish *N. furzeri*. Amputation site is indicated by red dashed line. **B** Chromogenic in situ hybridization of the injury-responsive gene *fstl1*. 1 dpa and 3 dpa tail fin tissues fixed with 4% PFA and probed for *fstl1.***C** ISH for *fstl1* in tail fins fixed with 4% PFA + formic acid. Brightfield images were taken with a stereomicroscope. Scale bars are 500 μm

Given the NAFA protocol’s superior preservation of delicate tissues in planaria, we next sought to determine if it can be adapted to study regeneration responses in other organisms. Current ISH protocols have performed poorly for probing gene expression changes in large whole-mount samples, particularly those involving the establishment of wound epidermis and a regeneration blastema in adults (e.g., the teleost caudal fin). The short-lived African killifish *Nothobranchius furzeri* can regenerate appendages and even organs such as the heart after injury, making them ideally suited to investigate tissue regeneration in adult animals [25, 26]. However, WISH experiments on the regenerating killifish tail fin can be difficult due to high variability and low signal to noise ratio [25]. To test whether the use of formic acid during fixation can facilitate robust WISH signal development, amputated killifish tail fins were fixed using 4% paraformaldehyde (PFA) with or without formic acid at 1 and 3 dpa (Fig. 8A). ISH for an early blastema gene *follistatin-like-1 (fstl1)* showed that the use of formic acid in the fixative increased the signal-to-noise ratio resulting in intense signal at the site of injury. In contrast, the *fstl1* signal in samples fixed without formic acid was masked by background noise (Fig. 8B, C). Similar results were observed for a blastema gene, *wnt10a*, in 3 dpa samples (Additional file 10: Fig. S10). These results demonstrate that adding formic acid to the fixative can enhance ISH signals in regenerating fish fins, facilitating global analysis of gene expression dynamics. Furthermore, it highlights the robustness of the NAFA protocol and shows that it can be easily adapted to a variety of tissues and organisms.

## Discussion

Preservation of external tissue layers is especially important for a research organism used to study regeneration, because stem cell proliferation and differentiation take place just beneath the wounding epidermis and form a blastema which grows to replace lost tissues [27]. Current ISH protocols facilitate probe penetration with harsh chemical treatments which damage delicate tissues, such as the ciliated epidermis in planarians and blastemas in both vertebrates and invertebrates. These same treatments can also damage or eliminate epitopes necessary for immunostainings. The new NAFA protocol addresses these shortcomings and allows for performing immunofluorescence and ISH on the same samples while preserving the delicate outer cellular layers of the planarian *S. mediterranea*. The use of formic acid fixative also enhanced ISH results in the regenerating tail fin of the African killifish *N. furzeri*. The greatly improved tissue integrity and increased signal to noise ratio provided by the NAFA protocol will enable researchers to investigate gene expression changes during wound healing and blastema formation.

The NAFA protocol, like the NA (Rompolas) protocol, is highly compatible with immunofluorescence. Both protocols use nitric acid during fixation, which is known to euthanize and flatten planarians while preserving the ciliated epidermis [11, 12, 28]. However, use of nitric acid alone is not sufficient to enable detection of gene expression by ISH. To develop a protocol that is compatible with both ISH and immunofluorescence, we explored the use of carboxylic acids, which are widely used in a variety of fixation approaches [29]. These methods are a subset of a broader class called coagulant fixatives which act by precipitating proteins instead of covalently crosslinking them [14]. Acid treatments enhance immunohistochemical studies by hydrolyzing crosslinks and potentially disrupting protein complexes, in a process known as antigen retrieval [30]. In contrast, the NAC protocol uses enzymatic proteinase K treatment to permeabilize the sample. While immunofluorescence signals can be generated from this method, these signals are much weaker at times than those produced by the NAFA or NA (Rompolas) methods, presumably due to the loss of target epitopes by enzymatic digestion. Furthermore, the harsh mucolytic NAC treatment tears the outer layers of the planarian body, making it difficult to use for studying fragile tissues such as the epidermis and regeneration blastema.

The NAFA protocol is also highly compatible with in situ hybridization, in stark contrast to the NA (Rompolas) protocol. Three main possibilities exist to account for this compatibility: (1) that samples fixed using the NAFA protocol are more permeable to riboprobes than samples fixed by the NA (Rompolas) protocol, (2) that RNA targets are more available to ISH probes than they are in other coagulating fixation conditions, or (3) that target RNA molecules are better preserved by NAFA than they are with harsher acid treatments. Below, we evaluate the likelihood of each of these three possibilities.

First, samples fixed with the NA (Rompolas) protocol are sufficiently permeabilized to allow antibodies to penetrate to internal structures detectable by immunofluorescence, yet in situ hybridization fails on these samples. While the structures of specific antisense mRNA probes are unknown, the relatively short probes used in this study still do not yield any appreciable signal with the NA (Rompolas) protocol. This suggests that sample permeability may not explain NAFA’s superior performance in ISH. Because size affects diffusion rate and riboprobe penetration, a systematic study with probes of varying lengths is necessary to assess permeabilization in samples fixed by each method. Second, relative to prolonged strong acid treatments, such as the NA (Rompolas) protocol, the proteins in NAFA samples will likely not be hydrolyzed to the same extent, and will also be crosslinked, two factors which would be expected to increase the size and complexity of proteins bound to and around RNA molecules. Since NAFA fixation likely leads target RNA molecules to be bound or surrounded by networks of crosslinked proteins, we hypothesize that increased RNA availability to probes is another unlikely explanation for the compatibility of NAFA with ISH. Third, compared to the NA (Rompolas) protocol, NAFA’s much briefer nitric acid treatment almost certainly results in less acid hydrolysis of RNA. Furthermore, the NAFA protocol includes EGTA to chelate calcium ions, as many RNase enzymes require these to digest RNA molecules [15]. Of the three possibilities for the NAFA protocol’s compatibility with ISH, we posit that preservation of RNA integrity is the most likely explanation.

The benefits of the NAFA protocol are likely due to the unique approach of simultaneously performing crosslinking and carboxylic acid treatments. As we devised this method, we tested three carboxylic acids for their performance in ISH and chose formic acid, which is chemically the smallest and simplest carboxylic. Formic acid is the strongest of the three acids tested in this study. It is unknown whether other untested carboxylic acids would perform better on ISH in planarians. However, for aliphatic carboxylic acids such as the ones tested here, increasing length of the carbon chain is inversely proportional to acid strength, so we expect other acids would be unlikely to produce the full benefits created by the formic acid treatment of the NAFA protocol. Furthermore, carboxylic acids with long aliphatic carbon chains have detergent-like properties, making them potentially unsuitable for fixing tissue samples.

The NAFA protocol can be used for preparing whole-mount planarian samples for immunofluorescence, ISH, and tissue sections for histological stainings like AFOG. The combination of using a carboxylic acid like formic acid in the fixative also improved ISH signal in the killifish tail fin indicating the ease of adapting this protocol for a wide variety of research organisms. Given the success of the NAFA protocol in traditional ISH protocol with long riboprobes, it is likely compatible with Hybridization Chain Reaction v3.0 (HCR) which uses multiple short RNA probes [31]. Future studies will determine the compatibility of NAFA fixation with HCR. Because it preserves the integrity of the ciliated epidermis in planarians, this method may be useful for the study of other samples with multiciliated cells, such as the lung epithelium, oviduct, and inner ear. Future work will explore the applicability of the NAFA protocol in a diverse array of samples and research organisms.

## Conclusions

We describe a fixation protocol using nitric acid and formic acid (NAFA) which preserves the fragile tissues such as the planarian regeneration blastema and epidermis. NAFA protocol is compatible with a variety of downstream assays such as in situ hybridization, immunofluorescence, and histological stainings. The protocol was also easily adapted to probe for gene expression in the regenerating killifish tail fin. Thus, the method promises to be broadly applicable for a variety of tissues and research organisms.

## Methods

### Animal husbandry

Asexual *Schmidtea mediterranea* planarians were grown in 1 × Montjuic water in recirculating systems or static cultures in Tupperware boxes at 20 °C [16, 32]. When maintained in static cultures 1 × Montjuic water was supplemented with gentamycin (50–100 µg/mL). Animals were fed with either beef liver chunks or puree, 1–3 times a week. Animals were starved for at least 1 week before use in experiments [16].

The inbred strain GRZ of the African turquoise killifish *Nothobranchius furzeri* were grown at 26 °C, and caudal fin amputation was carried out as described previously [25, 33]. All vertebrate work was performed according to the protocols approved by the Stowers Institute for Medical Research Institutional Animal Care and Use Committee.

### Riboprobe synthesis

Hapten-labeled antisense RNA probes were synthesized with a few modifications to the previously published protocol [9]. Up to 1 μg of PCR-amplified DNA templates were used for T7 based in vitro transcription reaction to generate antisense RNA sequences. Probes were synthesized for either 2 h or overnight at 37 °C in a thermocyler using digoxigenin (DIG), fluorescein, or DNP-labeling mix. Template DNA was degraded by incubating the reaction with RNase-free DNase for 45 min at 37 °C. Riboprobes were precipitated at − 80 °C for 1 h in 0.5 volumes of 7.5 M ammonium acetate and 2 volumes of ice-cold ethanol. RNA pellet was obtained by centrifugation at 14,000 rpm for 30 min at 4 °C. RNA pellet was washed in 75% ethanol and air dried before resuspending in 100 μL of deionized formamide. We generally used these riboprobes at 1:1000 dilution in ISH experiments.

### NA (Rompolas), NAC, and NAFA fixation

Fixation with NA (Rompolas) protocol was carried out as described before [34] with the following modifications: fixation with relaxant solution was carried out for 16 h at RT. Animals were washed in PBS and post-fixed with 4% paraformaldehyde in PBS for 10 min. Samples were permeabilized in 1% IGEPAL CA-360 for 10 min and washed with PBSTx prior to carrying out ISH or immunostaining experiments.

Animals were fixed using NAC protocol as described previously [9, 10]. Briefly, animals were euthanized in 5% NAC for 5 min and fixed in 4% formaldehyde for 45 min. Animals were dehydrated in methanol and stored in − 20 °C at least overnight and up to several months. When ready to use for the experiments, samples were rehydrated in PBSTx and bleached using formamide bleach for 2 h. Animals were permeabilized with proteinase K for 10 min and post-fixed with 4% formaldehyde for 10 min. Following two 10-min washes with PBSTx, samples were continued with either ISH or immunostaining procedures.

In NAFA fixation, animals were euthanized in NA solution and fixed in FA solution for 45 min. Following fixation, animals were dehydrated in methanol and stored in − 20 °C until ready for use. Animals were rehydrated and bleached in formamide bleach for 2 h before continuing with either ISH or immunostaining. The detailed step-by-step protocol for NAFA fixation is provided in Additional files 11–15. All the recipes for solutions used in the protocol are described in Additional file 16. All chemicals used in the study are listed in Additional file 17: Supplementary Table 1.

### ISH and immunostaining

Animals fixed with the three different methods were treated identically for ISH and immunostaining following previously published protocols [9, 10, 18, 35]. Fluorescently conjugated tyramides were synthesized from N-hydroxy-succinimidyl esters as previously described [36]. The detailed step-by-step protocols for ISH and immunostaining are provided in Supplementary Files 1A-1E.

### Histological sectioning and AFOG staining

WISH-stained animals were cryosectioned at 7 µm thickness as described previously [37]. For Acid Fuchsin Orange G (AFOG) staining, fixed samples were embedded in paraffin and processed into 10-μm-thick sections. AFOG staining was carried out as previously described [25].

### Imaging

Colorimetric WISH samples were imaged on Leica M205 stereo microscope. Fluorescent images were taken on a Zeiss confocal microscope or Nikon Spinning disk and processed in Fiji [38]. For Figs. 1 and 5, animals were mounted either dorsally or ventrally to capture surface ciliary patterns. H3P densities were determined from maximum intensity projections as described before [39]. H3P intensity was determined by the brightness of each focus identified by Fiji’s “Find maxima” function. Average *piwi-1* intensity was calculated from maximum intensity projections.

## Supplementary Information

Additional file 1: Supplementary Fig. S1. Epidermal integrity is preserved with NAFA protocol. Chromogenic in situ of *zpuf-6* (epidermal progenitor). Transverse histology section taken anterior to the pharynx. (A) Samples fixed with the NAC protocol. Black arrows indicate damage to the epidermal layer. (B) Samples fixed with the NAFA protocol. No disruptions to the epidermis are visible. Brightfield images were taken with a stereomicroscope. Scale bars: 100 μm.

Additional file 2: Supplementary Fig. S2. Different carboxylic acids tested in optimization of a new in situ protocol. Chromogenic WISH of *piwi-1*, *zpuf-6*, *pc2*, and *porcupine*. (A) NAC, (B) formic acid (4.8%), (C) acetic acid (4.9%) and (D) lactic acid (4.2%). Brightfield images were taken with a stereomicroscope. Scale bars: 100 μm.

Additional file 3: Supplementary Fig. S3. FISH signal intensities are comparable between NAFA and NAC protocols. (A) Mean intensity of *piwi-1* FISH signal was calculated from the max projections represented in Fig. 3. Plot shows box and whisker plot for three animals per condition. *P*-values were calculated with Student’s *t*-test.

Additional file 4: Supplementary Fig. S4. NAFA protocol yields brighter H3P signal without changing the density of dividing cells. (A) Comparison of H3P^+^ nuclei per square millimeter. (B) Comparison of mean fluorescence intensity of H3P^+^ nuclei from each animal. Box and whisker plots show median values and interquartile ranges. *N* = 3 animals per condition. *P*-values for (A) and (B) were calculated with Student’s *t*-test. (C) Representative images of H3P stainings. Top row: all images shown with same brightness/contrast settings, optimized to NAFA. Bottom row: each image shown with custom settings. Images are max projections of confocal stacks, scale bars: 50 μm.

Additional file 5: Supplementary Fig. S5. Consistent labeling of muscle fibers by the NAFA protocol. (A) Max projections of confocal image stacks of animals immunostained with the muscle antibody Smed-6G10. Images are arranged with anterior to the left for all animals. For rows 1–2 the dorsal surface is visible, while for rows 3–6 the ventral surface and mouth are visible. All six animals processed for each condition are shown, scale bars: 200 μm.

Additional file 6: Supplementary Fig. S6. Immunostaining of internal structures by the NAFA protocol. Maximum intensity projections of confocal image substacks to specifically visualize external and internal structures. (A) 40 × magnification image of the body wall musculature stained by Smed-6G10. Scale bars: 50 μm. (B) Upper: Maximum intensity projection of 3 z-stacks of whole-mount immunostaining showing gut musculature. Scale bars: 200 μm. Lower: Maximum intensity projection of sub-stacks of 40 × magnification image of gut musculature in tail stripe region, posterior to pharynx. Scale bars 50 μm. (C) Maximum intensity projection of top 15 microns of 40 × magnification immunofluorescence images of the ventral ciliated epidermis. Upper: anti-acetylated tubulin (gray), lower: merge with DAPI (blue). (D) Similar to C), for different substacks to highlight protonephridia staining by anti-acetylated tubulin. All scale bars for C) and D) are 50 μm.

Additional file 7: Supplementary Fig. S7 Densities of mitotic cells are comparable between NAFA and NAC protocols. Number of H3P + nuclei were counted and divided by the area of the worm to obtain density. These number are from the max projection images represented in Fig. 5. Each dot represents an animal. *P*-values were calculated with Student’s *t*-test.

Additional file 8: Supplementary Fig. S8. NAFA protocol maintains epidermal and blastema integrity during regeneration. (A) DAPI staining. (B) FISH of *zpuf-6* and DAPI staining. (C) FISH of *zpuf-6* and immunostaining of anti-acetylated tubulin (cilia). (B and C) White arrows show the affected epidermal layer and red arrows at the blastema show epidermal integrity during regeneration. Maximum intensity projection of confocal images (40X). Ant: anterior and Post: posterior. Regenerating trunk fragments. Scale bars are 50 μm.

Additional file 9: Supplementary Fig. S9. NAFA protocol is compatible with histological staining and maintains epidermal integrity during regeneration. (A) 8 hpa longitudinal sections stained with AFOG. Magnified images of the area around the wound marked by yellow dotted box are shown. (B) 4 dpa longitudinal sections stained with AFOG. Areas of the zoomed in images are highlighted by dashed boxes. Red arrows mark the epidermis. Brightfield images were taken with a compound microscope. Scale bars for whole-mounts are 100 μm, 50 μm for insets.

Additional file 10: Supplementary Fig. S10. Expression of *wnt10a* at the blastema is better observed when the tissue is fixed in the presence of formic acid. (A) Chromogenic in situ hybridization for the injury-responsive gene *wnt10a.* 3 dpa tail fins fixed with or without formic acid and probed for *wnt10a.* Amputation site is indicated by red dashed line. Brightfield images were taken with a stereomicroscope. Scale bars are 500 μm.

Additional file 11: Detailed step by step protocol describing colorimetric WISH with NAFA fixation.

Additional file 12: Detailed step by step protocol describing FISH using the NAFA fixation.

Additional file 13: Detailed step by step protocol describing FISH and immunostaining protocol with NAFA fixation.

Additional file 14: Detailed step by step protocol describing whole mount immunofluorescence staining using the NAFA fixation.

Additional file 15: Detailed step by step protocol for colorimetric WISH on killifish fins using the NAFA protocol.

Additional file 16: Details of solutions used for fixation, WISH, and immunofluorescence.

Additional file 17: Supplementary Table 1 – Vendor information and catalog numbers for all the reagents required to carry out NAFA fixation, WISH, and immunofluorescence.

## Data Availability

All the data generated and analyzed during this study are included in the published article. The raw data are also available at the Stowers Original Data Repository, http://www.stowers.org/research/publications/libpb-2493.

## Abbreviations

WISH: Whole-mount in situ hybridization
ISH: In situ hybridization
NAFA: Nitric Acid/Formic Acid
NAC: N-Acetyl cysteine
EGTA: Ethylene glycol-bis (β-aminoethyl ether)-N,N,N′,N′-tetraacetic acid
FISH: Fluorescent in situ hybridization
H3P: Serine-10 phosphorylated form of histone H3
hpa: Hours post amputation
dpa: Days post amputation
AFOG: Acid Fuchsin Orange G staining
PFA: Paraformaldehyde
