# Supplementary figures and images for: A powerful and versatile new fixation protocol for immunostaining and in situ hybridization that preserves delicate tissues
Source: BMC Biol. 2024 Nov 4;22:252. doi: 10.1186/s12915-024-02052-3 (PMC11533299; doi:10.1186/s12915-024-02052-3)

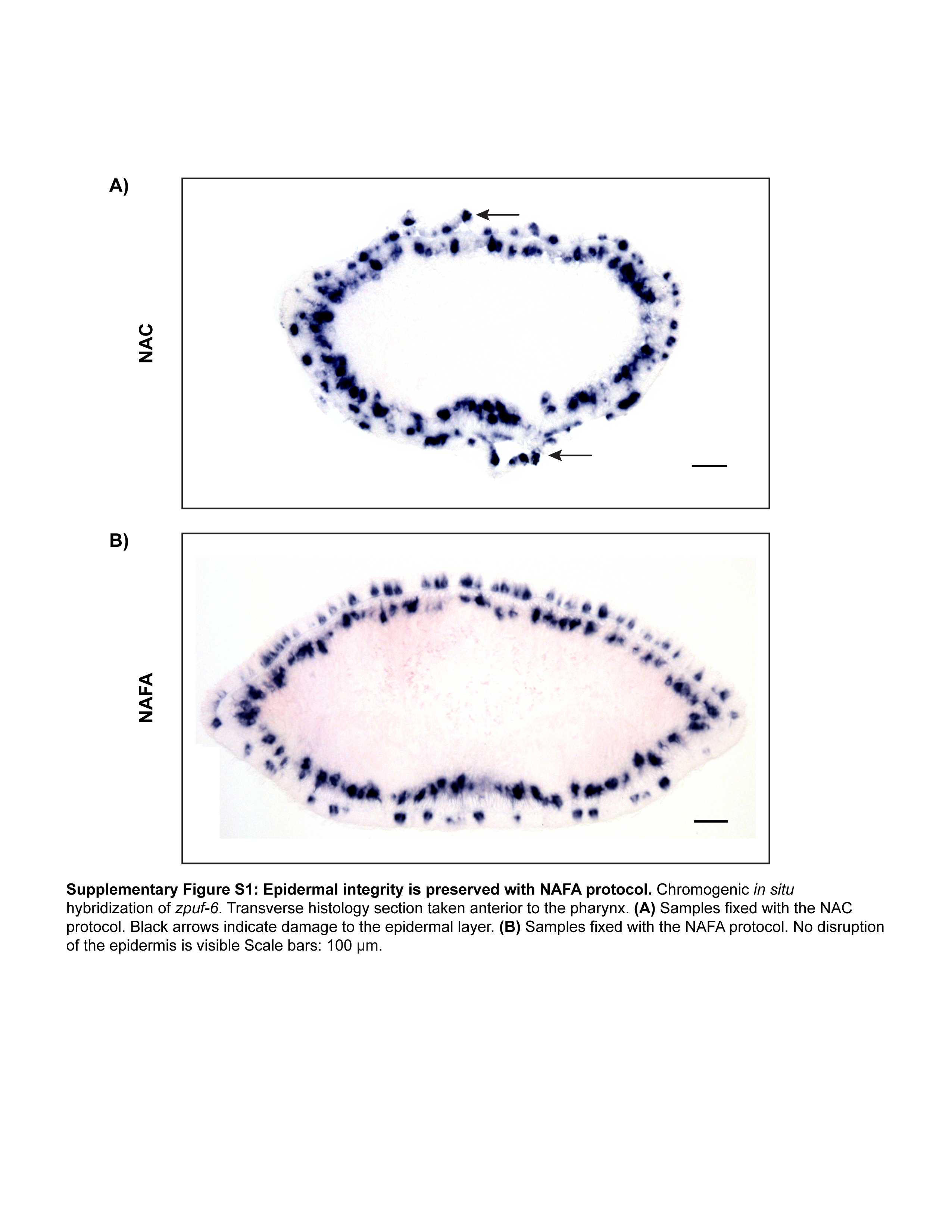

Supplement: Supplementary file 1 — Additional file 1: Supplementary Fig. S1. Epidermal integrity is preserved with NAFA protocol. Chromogenic in situ of zpuf-6 (epidermal progenitor). Transverse histology section taken anterior to the pharynx. (A) Samples fixed with the NAC protocol. Black arrows indicate damage to the epidermal layer. (B) Samples fixed with the NAFA protocol. No disruptions to the epidermis are visible. Brightfield images were taken with a stereomicroscope. Scale bars: 100 μm. [file 12915_2024_2052_MOESM1_ESM.tif]

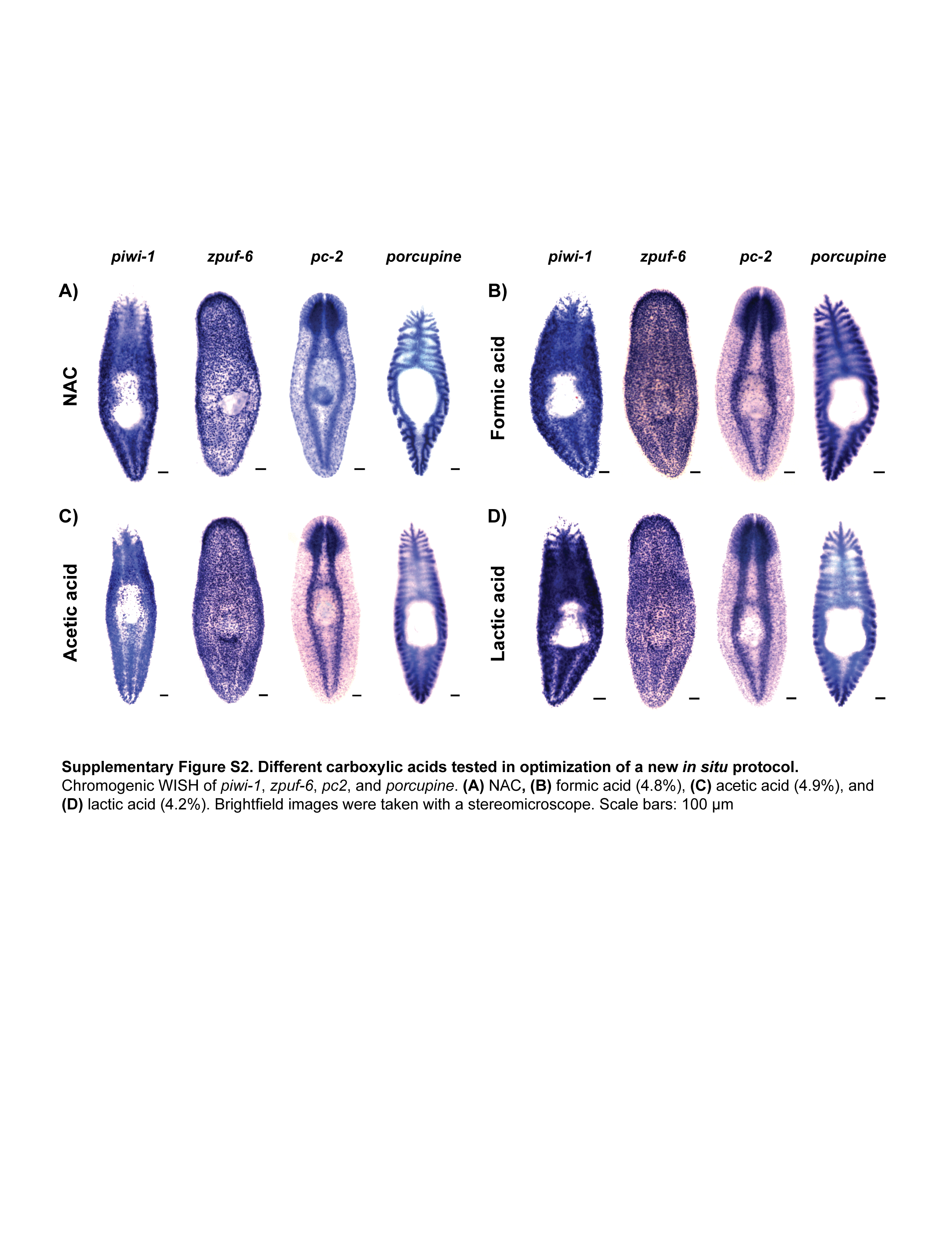

Supplement: Supplementary file 2 — Additional file 2: Supplementary Fig. S2. Different carboxylic acids tested in optimization of a new in situ protocol. Chromogenic WISH of piwi-1, zpuf-6, pc2, and porcupine. (A) NAC, (B) formic acid (4.8%), (C) acetic acid (4.9%) and (D) lactic acid (4.2%). Brightfield images were taken with a stereomicroscope. Scale bars: 100 μm. [file 12915_2024_2052_MOESM2_ESM.tif]

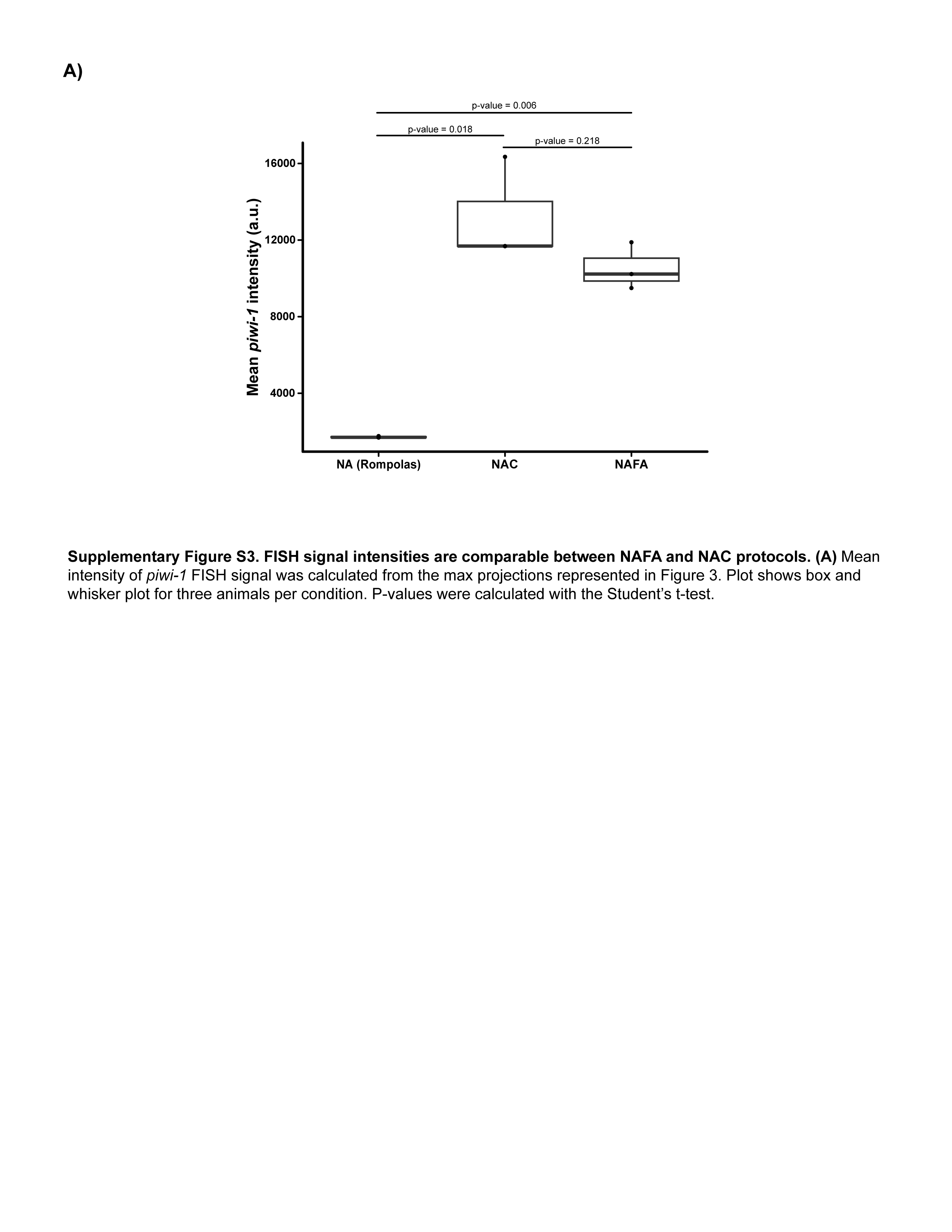

Supplement: Supplementary file 3 — Additional file 3: Supplementary Fig. S3. FISH signal intensities are comparable between NAFA and NAC protocols. (A) Mean intensity of piwi-1 FISH signal was calculated from the max projections represented in Fig. 3. Plot shows box and whisker plot for three animals per condition. P-values were calculated with Student’s t-test. [file 12915_2024_2052_MOESM3_ESM.tif]

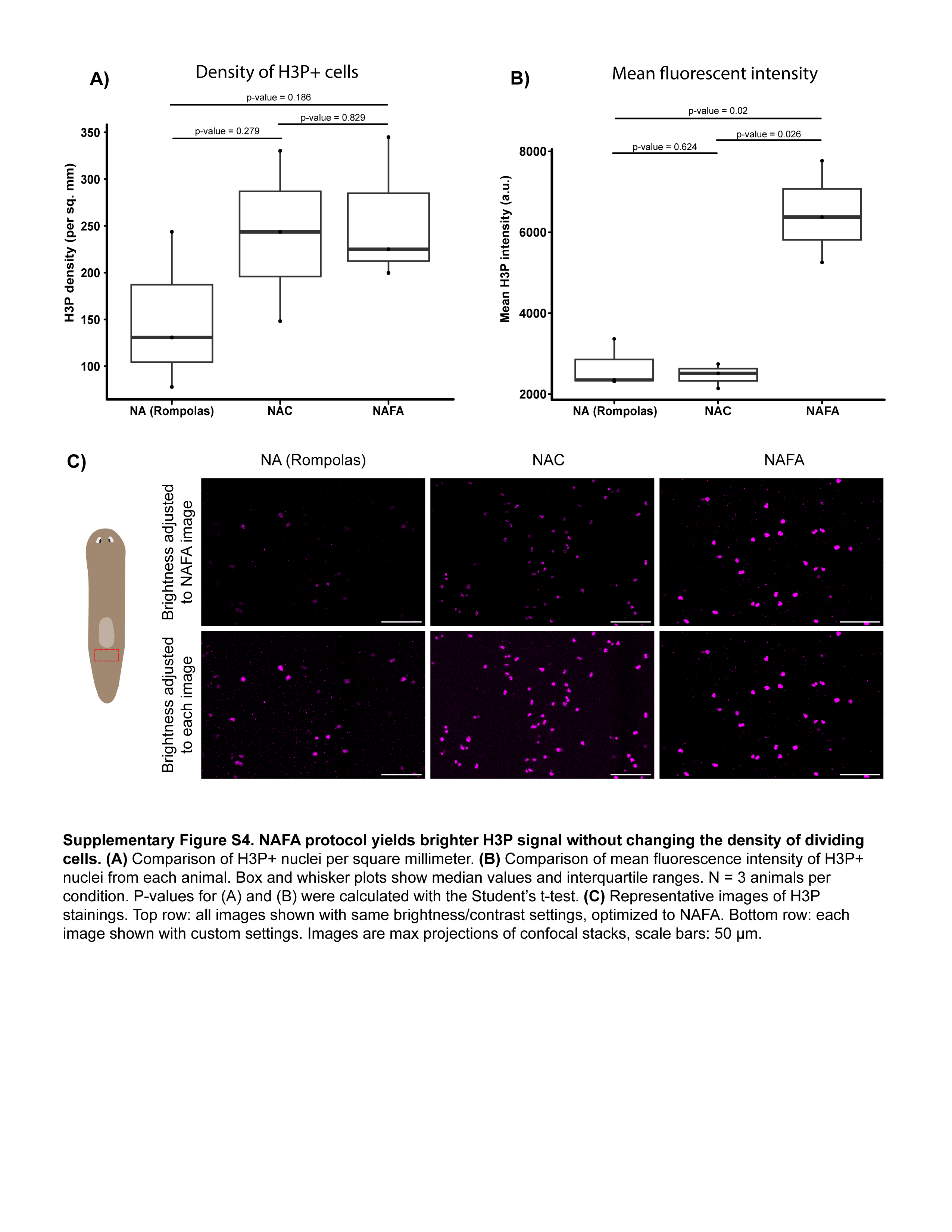

Supplement: Supplementary file 4 — Additional file 4: Supplementary Fig. S4. NAFA protocol yields brighter H3P signal without changing the density of dividing cells. (A) Comparison of H3P+ nuclei per square millimeter. (B) Comparison of mean fluorescence intensity of H3P+ nuclei from each animal. Box and whisker plots show median values and interquartile ranges. N = 3 animals per condition. P-values for (A) and (B) were calculated with Student’s t-test. (C) Representative images of H3P stainings. Top row: all images shown with same brightness/contrast settings, optimized to NAFA. Bottom row: each image shown with custom settings. Images are max projections of confocal stacks, scale bars: 50 μm. [file 12915_2024_2052_MOESM4_ESM.tif]

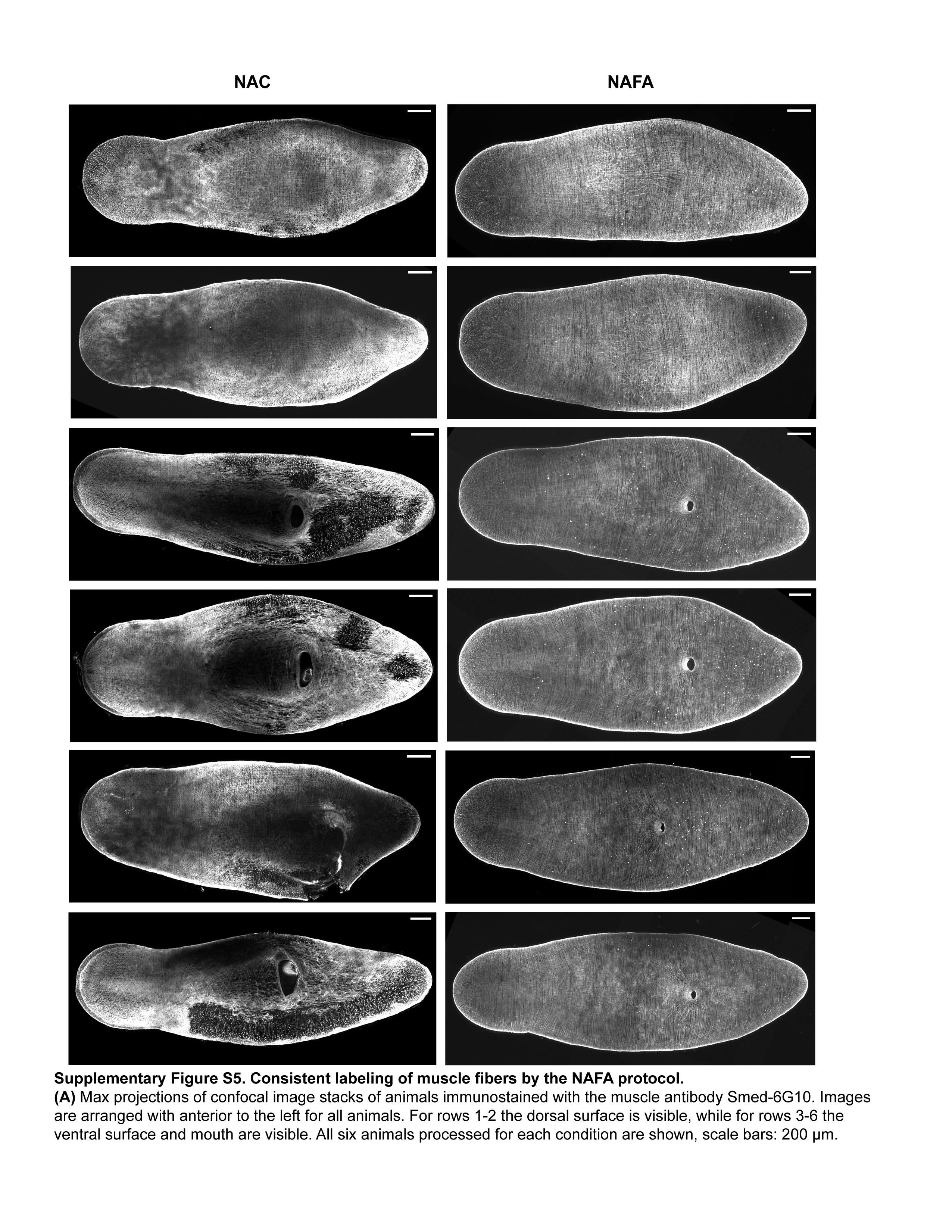

Supplement: Supplementary file 5 — Additional file 5: Supplementary Fig. S5. Consistent labeling of muscle fibers by the NAFA protocol. (A) Max projections of confocal image stacks of animals immunostained with the muscle antibody Smed-6G10. Images are arranged with anterior to the left for all animals. For rows 1–2 the dorsal surface is visible, while for rows 3–6 the ventral surface and mouth are visible. All six animals processed for each condition are shown, scale bars: 200 μm. [file 12915_2024_2052_MOESM5_ESM.tif]

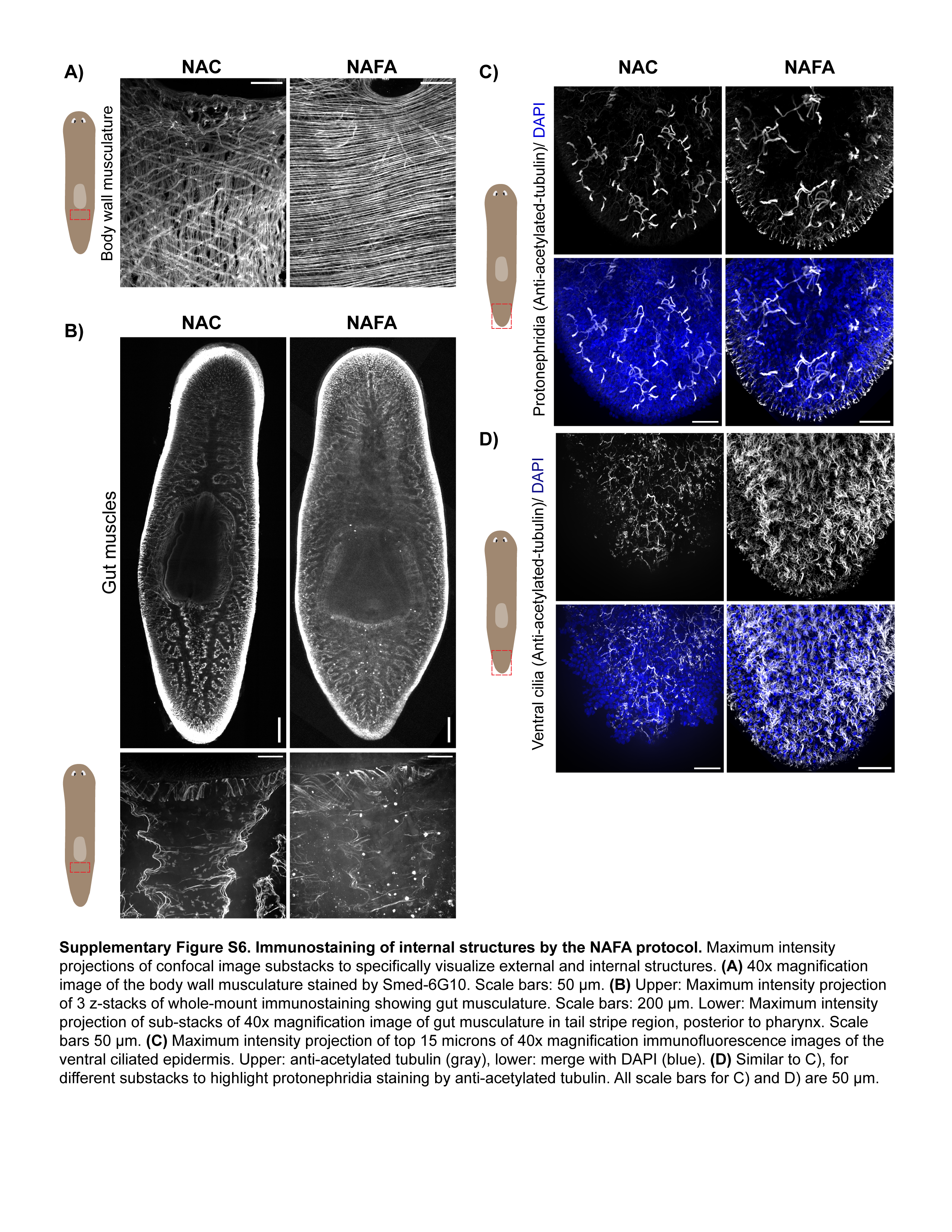

Supplement: Supplementary file 6 — Additional file 6: Supplementary Fig. S6. Immunostaining of internal structures by the NAFA protocol. Maximum intensity projections of confocal image substacks to specifically visualize external and internal structures. (A) 40 × magnification image of the body wall musculature stained by Smed-6G10. Scale bars: 50 μm. (B) Upper: Maximum intensity projection of 3 z-stacks of whole-mount immunostaining showing gut musculature. Scale bars: 200 μm. Lower: Maximum intensity projection of sub-stacks of 40 × magnification image of gut musculature in tail stripe region, posterior to pharynx. Scale bars 50 μm. (C) Maximum intensity projection of top 15 microns of 40 × magnification immunofluorescence images of the ventral ciliated epidermis. Upper: anti-acetylated tubulin (gray), lower: merge with DAPI (blue). (D) Similar to C), for different substacks to highlight protonephridia staining by anti-acetylated tubulin. All scale bars for C) and D) are 50 μm. [file 12915_2024_2052_MOESM6_ESM.tif]

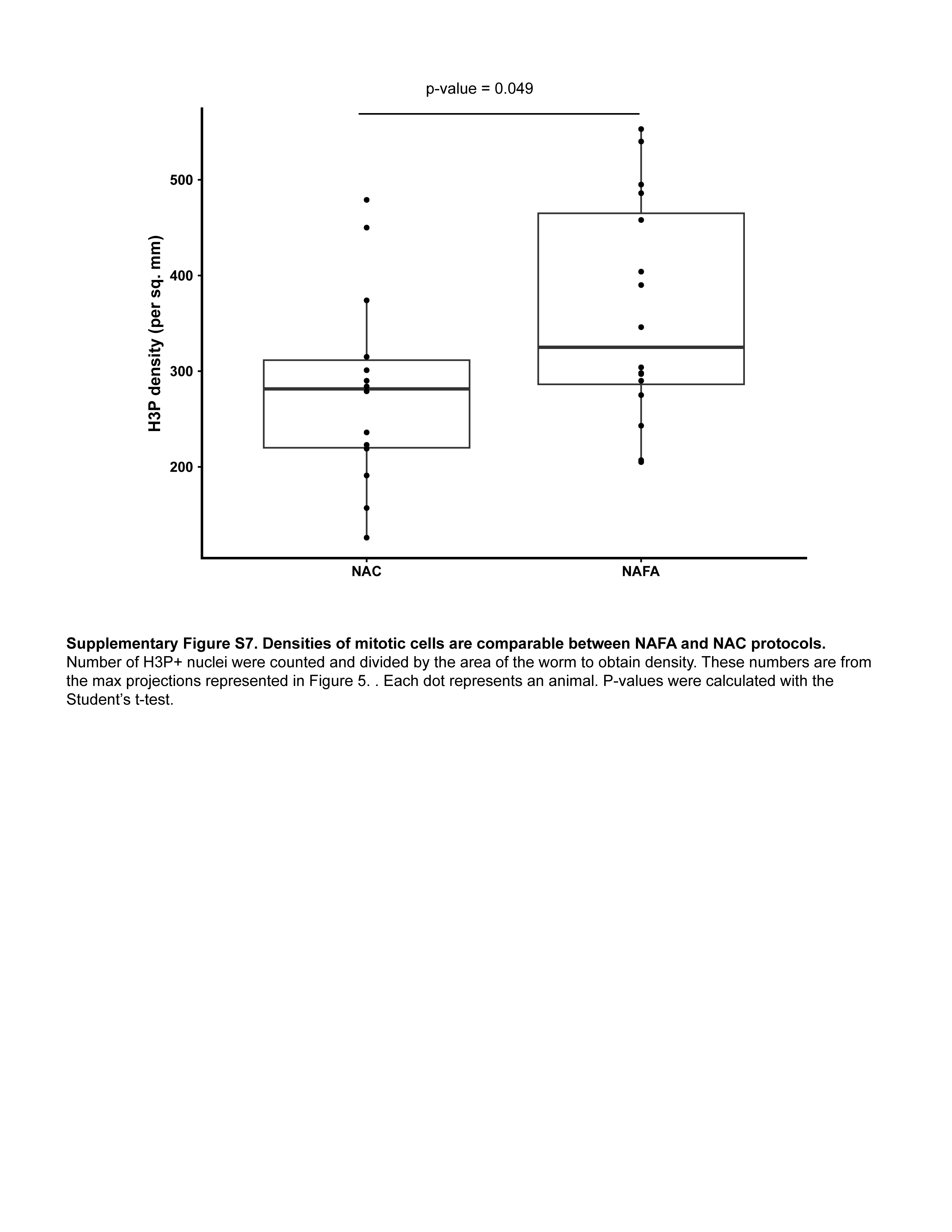

Supplement: Supplementary file 7 — Additional file 7: Supplementary Fig. S7 Densities of mitotic cells are comparable between NAFA and NAC protocols. Number of H3P + nuclei were counted and divided by the area of the worm to obtain density. These number are from the max projection images represented in Fig. 5. Each dot represents an animal. P-values were calculated with Student’s t-test. [file 12915_2024_2052_MOESM7_ESM.tif]

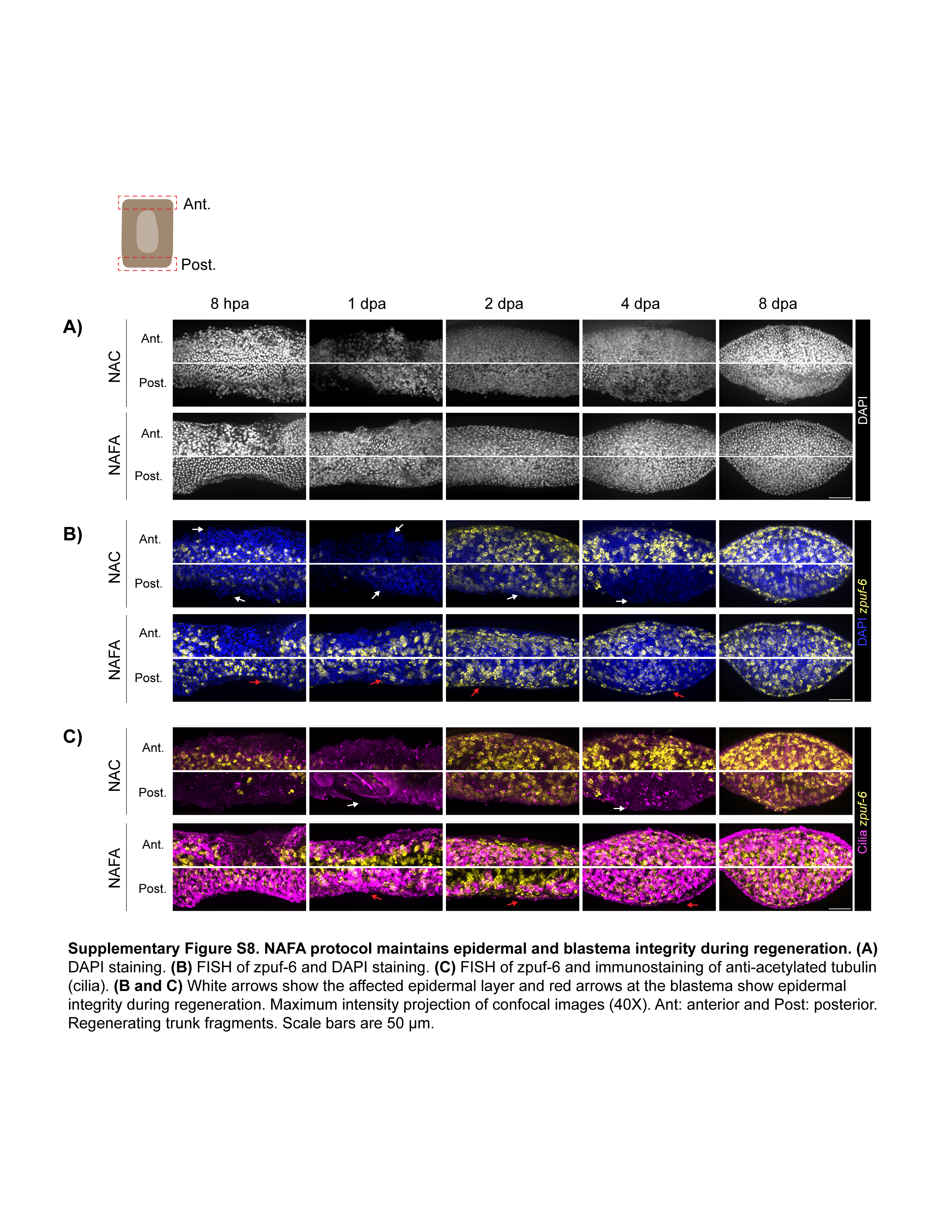

Supplement: Supplementary file 8 — Additional file 8: Supplementary Fig. S8. NAFA protocol maintains epidermal and blastema integrity during regeneration. (A) DAPI staining. (B) FISH of zpuf-6 and DAPI staining. (C) FISH of zpuf-6 and immunostaining of anti-acetylated tubulin (cilia). (B and C) White arrows show the affected epidermal layer and red arrows at the blastema show epidermal integrity during regeneration. Maximum intensity projection of confocal images (40X). Ant: anterior and Post: posterior. Regenerating trunk fragments. Scale bars are 50 μm. [file 12915_2024_2052_MOESM8_ESM.tif]

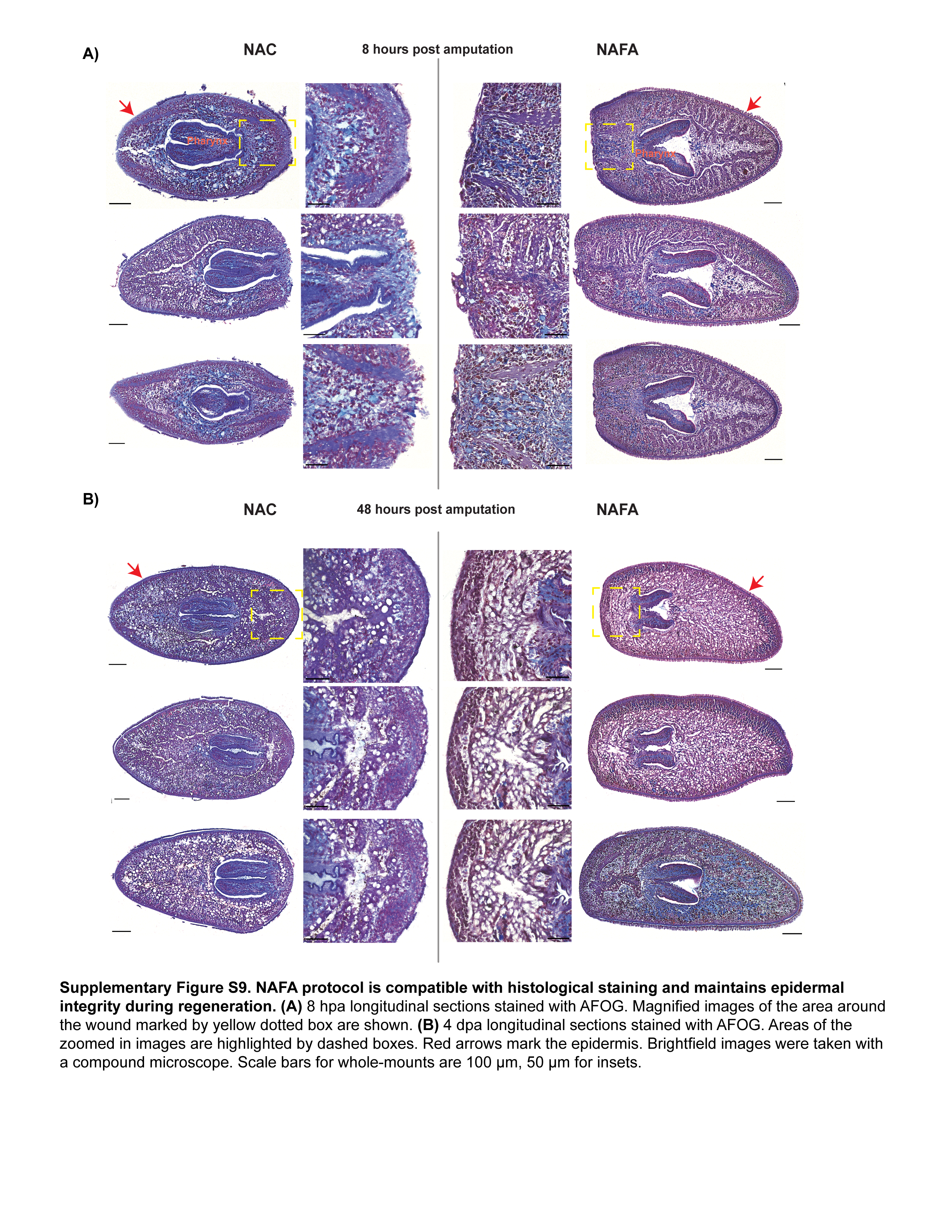

Supplement: Supplementary file 9 — Additional file 9: Supplementary Fig. S9. NAFA protocol is compatible with histological staining and maintains epidermal integrity during regeneration. (A) 8 hpa longitudinal sections stained with AFOG. Magnified images of the area around the wound marked by yellow dotted box are shown. (B) 4 dpa longitudinal sections stained with AFOG. Areas of the zoomed in images are highlighted by dashed boxes. Red arrows mark the epidermis. Brightfield images were taken with a compound microscope. Scale bars for whole-mounts are 100 μm, 50 μm for insets. [file 12915_2024_2052_MOESM9_ESM.tif]

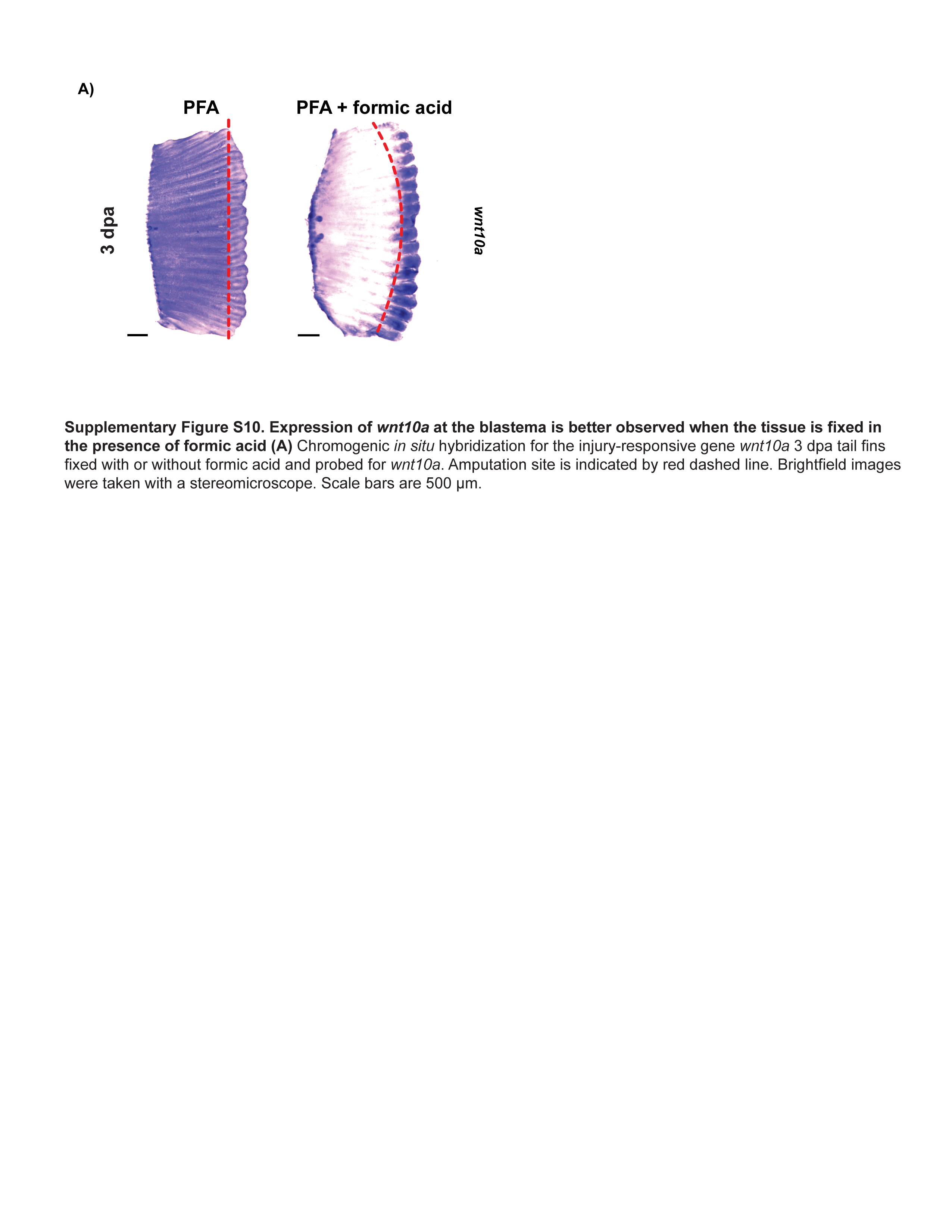

Supplement: Supplementary file 10 — Additional file 10: Supplementary Fig. S10. Expression of wnt10a at the blastema is better observed when the tissue is fixed in the presence of formic acid. (A) Chromogenic in situ hybridization for the injury-responsive gene wnt10a. 3 dpa tail fins fixed with or without formic acid and probed for wnt10a. Amputation site is indicated by red dashed line. Brightfield images were taken with a stereomicroscope. Scale bars are 500 μm. [file 12915_2024_2052_MOESM10_ESM.tif]
