# Supplementary material for: A powerful and versatile new fixation protocol for immunostaining and in situ hybridization that preserves delicate tissues
Source: BMC Biol. 2024 Nov 4;22:252. doi: 10.1186/s12915-024-02052-3 (PMC11533299; doi:10.1186/s12915-024-02052-3)
Supplement: Supplementary file 11 — Additional file 11: Detailed step by step protocol describing colorimetric WISH with NAFA fixation. [file 12915_2024_2052_MOESM11_ESM.pdf]

Colorimetric WISH protocol using Nitric acid / formic acid (NAFA) fixation for planarians

Note: All the steps are carried out with animals being nutated/rocked at room temperature unless stated otherwise.

1. Transfer planarians (up to ~5 mm in size) starved for at least for one week to either 1.5 mL tubes or 15 mL tubes for processing up to 20 or 100 animals, respectively.

2. Replace planarian water with NA solution for 1-2 minutes. During this treatment agitate animals vigorously by inverting the tubes a few times. NA solution has nitric acid and magnesium sulfate which helps anesthetize (relax) and euthanize the animals prior to fixation.
   Note: This treatment should not go beyond 5 minutes.

3. Replace the NA solution with FA solution and incubate the animals in this solution for 40 minutes to 1 hour.

4. Remove the FA solution and wash twice in 1X PBS for 10 minutes each.

5. Following the 1X PBS washes, wash animals in 50% methanol in 1X PBS for 10 minutes.

6. Replace the 50% methanol in 1X PBS with 100% methanol and incubate for 10 minutes to allow thorough dehydration.

7. Replace the solution with fresh 100% methanol and store in -20 °C for at least one hour or until ready to use.

8. When ready to use the fixed specimens, replace the 100% methanol with 50% methanol in 1X PBS for 10 minutes.

9. Once completed, replace the 50% methanol with 1X PBS for 10 minutes.

10. Bleach animals under direct light in formamide bleach solution for 2 hours.

11. Rinse the animals twice for 10 minutes each in PBSTx (0.3% - 0.5% Triton).

Following this step, directly proceed to *in situ* hybridization. There is no proteinase K treatment.

***in situ* hybridizations**

12. Replace PBSTx (0.3% - 0.5% Triton) with 1:1 (PBSTx:PreHybe) solution for 5 minutes.

13. Incubate animals in Pre-Hybe solution for 2 hours at 56 °C.

14. Replace Pre-Hybe with riboprobe mix (riboprobe(s) in hybridization buffer) for ≥16 hours at 56 °C. Riboprobes are generally used at 1:1000 dilution and can be heat denatured in hybridization buffer at 70 °C for 3 minutes prior to use.

15. Carry out post hybridization washes at 56 °C

    a. Wash with Wash Hybe two times for 30 minutes each.
    b. Wash with 1:1 mix of Wash Hybe:2X SSC (+ 0.1% Tween or Triton) two times for 30 minutes each.
    c. Wash three times with 2X SSC (+ 0.1% Tween or Triton) for 20 minutes each.
    d. Wash three times with 0.2X SSC (+ 0.1% Tween or Triton) for 20 minutes each.

16. Once post-hybridization washes are completed, wash animals three times with MABT, for 10 minutes each at room temperature.

17. Block with 5% filtered horse serum + 0.5% filtered Roche Western Blocking Reagent (RWBR) in MABT for 1-2 hours.

18. Incubate the samples overnight at room temperature, with appropriate antibody, diluted in blocking solution.

    We regularly use:

    a. Anti-DIG-AP at 1:2000 dilution
    b. Anti-DNP-AP at 1:1000 dilution

19. Wash animals 6 times in MABT for 20 minutes each.

NBT/BCIP based colorimetric signal development

20. After washing out the antibody with MABT (step 19), incubate in AP buffer for 1-2 minutes.

21. Replace the AP buffer with the EQ buffer for 10 minutes.

22. Incubate the animals in DEV buffer + NBT/BCIP till the signal is developed to appropriate levels.

23. Wash away NBT/BCIP by rinsing the samples two to three times in 1X PBS.

24. Post fix the samples in 4% formaldehyde in PBSTx (0.3% Triton) for 20 – 30 minutes.

25. Incubate the animals in 100% ethanol to clear the background.

26. Rinse the animals with 50% ethanol in 1X PBS for 5 minutes.

27. Wash the samples in 1X PBS for 5-10 minutes or until they sink.

28. Rinse the samples a couple more times in 1X PBS.

29. Clear in 80% glycerol or 75% Scale A2 for 1-2 days.
