# Supplementary material for: A powerful and versatile new fixation protocol for immunostaining and in situ hybridization that preserves delicate tissues
Source: BMC Biol. 2024 Nov 4;22:252. doi: 10.1186/s12915-024-02052-3 (PMC11533299; doi:10.1186/s12915-024-02052-3)
Supplement: Supplementary file 13 — Additional file 13: Detailed step by step protocol describing FISH and immunostaining protocol with NAFA fixation. [file 12915_2024_2052_MOESM13_ESM.pdf]

Fluorescent WISH and immunostaining protocol using Nitric acid / formic acid (NAFA) fixation for planarians

Note: All the steps are carried out with animals being nutated/rocked at room temperature unless stated otherwise.

1. Transfer planarians (up to ~5 mm in size) starved for at least for one week to either 1.5 mL tubes or 15 mL tubes for processing up to 20 or 100 animals, respectively.

2. Replace planarian water with NA solution for 1-2 minutes. During this treatment agitate animals vigorously by inverting the tubes a few times. NA solution has nitric acid and magnesium sulfate which helps anesthetize (relax) and euthanize the animals prior to fixation.
   Note: This treatment should not go beyond 5 minutes.

3. Replace the NA solution with FA solution and incubate the animals in this solution for 40 minutes to 1 hour.

4. Remove the FA solution and wash twice in 1X PBS for 10 minutes each.

5. Following the 1X PBS washes, wash animals in 50% methanol in 1X PBS for 10 minutes.

6. Replace the 50% methanol in 1X PBS with 100% methanol and incubate for 10 minutes to allow thorough dehydration.

7. Replace the solution with fresh 100% methanol and store in -20 °C for at least one hour or until ready to use.

8. When ready to use the fixed specimens, replace the 100% methanol with 50% methanol in 1X PBS for 10 minutes.

9. Once completed, replace the 50% methanol with 1X PBS for 10 minutes.

10. Bleach animals under direct light in formamide bleach solution for 2 hours.

11. Rinse the animals twice for 10 minutes each in PBSTx (0.3% - 0.5% Triton).

Following this step, directly proceed to *in situ* hybridization. There is no proteinase K treatment.

***in situ* hybridizations**

12. Replace PBSTx (0.3% - 0.5% Triton) with 1:1 (PBSTx:PreHybe) solution for 5 minutes.

13. Incubate animals in Pre-Hybe solution for 2 hours at 56 °C.

14. Replace Pre-Hybe with riboprobe mix (riboprobe(s) in hybridization buffer) for ≥16 hours at 56 °C. Riboprobes are generally used at 1:1000 dilution and can be heat denatured in hybridization buffer at 70 °C for 3 minutes prior to use.

15. Carry out post hybridization washes at 56 °C

    a. Wash with Wash Hybe two times for 30 minutes each.
    b. Wash with 1:1 mix of Wash Hybe:2X SSC (+ 0.1% Tween or Triton) two times for 30 minutes each.
    c. Wash three times with 2X SSC (+ 0.1% Tween or Triton) for 20 minutes each.
    d. Wash three times with 0.2X SSC (+ 0.1% Tween or Triton) for 20 minutes each.

16. Once post-hybridization washes are completed, wash animals three times with MABT, for 10 minutes each at room temperature.

17. Block with 5% filtered horse serum + 0.5% filtered Roche Western Blocking Reagent (RWBR) in MABT for 1-2 hours.

18. Incubate the samples overnight at room temperature, with appropriate antibody, diluted in blocking solution.

    We regularly use:

    a. Anti-DIG-POD at 1:1000 dilution
    b. Anti-Fluorescein-POD at 1:3000 dilution
    c. Anti DNP-HRP at 1:1000 dilution

19. Wash animals 6 times in MABT for 20 minutes each.

Tyramide-based fluorescent signal development

20. For developing fluorescent signal, preincubate the samples with tyramide in borate buffer for 15 minutes.
    a. FAM tyramide at 1:2000 dilution for DIG- or DNP-labeled probe and 1:5000 dilution for Fluorescein-labeled probe
    b. Cy3 tyramide at 1:1000 dilution
    c. Rhodmaine tyramide at 1:5000 dilution
    d. Cy5 tyramide at 1:1000 dilution

21. Carry out the tyramide reaction by adding 0.006% hydrogen peroxide in borate buffer for 45 minutes.
    Make fresh 1% H_2O_2 stock solution (966 ul of borate buffer and 33 ul of 30% H_2O_2). Add 6ul of 1% H2O2 solution to every 1ml of tyramide development solution.

22. Stop the reaction by washing out the samples twice in PBSTw (0.3% Tween) for 10 minutes each.

23. If developing a second probe –

    a. Kill the peroxidase activity by treating with 200mM sodium azide in PBSTw (0.3% Tween) for at least 1 hour.
    b. Wash 6 times with PBSTw (0.3% Tween) for 20 minutes each.
    c. Wash 4 times with MABT for 10 minutes each.
    d. Return to step 18 to develop the second probe.

24. If performing immunostaining skip to immunostaining protocol.

25. *Optional:* Post-fix the samples in 4% formaldehyde in PBSTx (0.3% Triton) for 20-30 minutes.

    Note: If performing immunostaining do not post fix at this stage.

26. Clear the samples for 1-2 days in 20% Scale A2 + DABCO.

Immunostaining

Antibody	Dilution	Source	Supplier	Catalog Number
Anti – H3P (S10+T11)	1:500 – 1:1000	Rabbit	Abcam	ab32107
Anti – acetylated tubulin	1:1000	Rabbit	Cell Signaling	#5335
Anti – acetylated tubulin	1:1000	Mouse	Sigma	T7451
6G10-2C7 (muscle antibody)	1:200 – 1:400	Mouse	Developmental Studies Hybridoma Bank (DSHB)	6G10-2C7
Anti-rabbit, Alexa Fluor 555	1:500 – 1:1000	Goat	Abcam	150086
Anti-rabbit, Alexa Fluor 647	1:500 – 1:1000	Goat	Abcam	150083
Anti-mouse, Alexa Fluor 555	1:500 – 1:1000	Goat	Abcam	150118
Anti-mouse, Alexa Fluor 647	1:500 – 1:1000	Goat	Abcam	150119

Note: If performing immunostaining on samples after developing *in situ* hybridization signal then start by blocking with 5% goat serum (step 1).

1. Fixed and bleached samples are blocked with 5% goat serum in PBSTw (0.3% - 0.5% Tween) for 1-2 hours.

2. Incubate the samples overnight with primary antibody in blocking solution (5% goat serum in PBSTw (0.3% - 0.5% Tween).

3. Wash the samples six times in PBSTw (0.3% - 0.5% Tween) for 20 minutes each.

4. Incubate overnight with appropriate secondary antibody (1:500 -1:1000) in blocking solution.

   Note: DAPI (0.2 ug/mL – 1 ug/mL) can be added at this step.

5. Wash the samples six times in PBSTw (0.3% - 0.5% Tween) for 20 minutes each.

6. Optional: Post-fix the samples in 4% formaldehyde in PBSTx (0.3% Triton) for 20 – 30 minutes.

7. Clear the samples for 1-2 days in 20% Scale A2 + DABCO.

   Note: We have not observed any significance difference in immunostaining when PBSTx (0.3% - 0.5% Triton) is used in place of PBSTw (0.3% - 0.5% Tween)
