# Supplementary material for: A powerful and versatile new fixation protocol for immunostaining and in situ hybridization that preserves delicate tissues
Source: BMC Biol. 2024 Nov 4;22:252. doi: 10.1186/s12915-024-02052-3 (PMC11533299; doi:10.1186/s12915-024-02052-3)
Supplement: Supplementary file 14 — Additional file 14: Detailed step by step protocol describing whole mount immunofluorescence staining using the NAFA fixation. [file 12915_2024_2052_MOESM14_ESM.pdf]

Whole mount immunostaining protocol using Nitric acid / formic acid (NAFA) fixation for planarians

Note: All the steps are carried out with animals being nutated/rocked at room temperature unless stated otherwise.

1. Transfer planarians (up to ~5 mm in size) starved for at least for one week to either 1.5 mL tubes or 15 mL tubes for processing up to 20 or 100 animals, respectively.

2. Replace planarian water with NA solution for 1-2 minutes. During this treatment agitate animals vigorously by inverting the tubes a few times. NA solution has nitric acid and magnesium sulfate which helps anesthetize (relax) and euthanize the animals prior to fixation.
   Note: This treatment should not go beyond 5 minutes.

3. Replace the NA solution with FA solution and incubate the animals in this solution for 40 minutes to 1 hour.

4. Remove the FA solution and wash twice in 1X PBS for 10 minutes each.

5. Following the 1X PBS washes, wash animals in 50% methanol in 1X PBS for 10 minutes.

6. Replace the 50% methanol in 1X PBS with 100% methanol and incubate for 10 minutes to allow thorough dehydration.

7. Replace the solution with fresh 100% methanol and store in -20 °C for at least one hour or until ready to use.

8. When ready to use the fixed specimens, replace the 100% methanol with 50% methanol in 1X PBS for 10 minutes.

9. Once completed, replace the 50% methanol with 1X PBS for 10 minutes.

10. Bleach animals under direct light in formamide bleach solution for 2 hours.

    Formamide bleach solution: 1% formamide, 6% hydrogen peroxide in PBSTx (0.3% - 0.5% Triton).

11. Rinse the animals twice for 10 minutes each in PBSTx (0.3% - 0.5% Triton).

Immunostaining

Antibody	Dilution	Source	Supplier	Catalog Number
Anti – H3P (S10+T11)	1:500 – 1:1000	Rabbit	Abcam	ab32107
Anti – acetylated tubulin	1:1000	Rabbit	Cell Signaling	#5335
Anti – acetylated tubulin	1:1000	Mouse	Sigma	T7451
6G10-2C7 (muscle antibody)	1:200 – 1:400	Mouse	Developmental Studies Hybridoma Bank (DSHB)	6G10-2C7
Anti-rabbit, Alexa Fluor 555	1:500 – 1:1000	Goat	Abcam	150086
Anti-rabbit, Alexa Fluor 647	1:500 – 1:1000	Goat	Abcam	150083
Anti-mouse, Alexa Fluor 555	1:500 – 1:1000	Goat	Abcam	150118
Anti-mouse, Alexa Fluor 647	1:500 – 1:1000	Goat	Abcam	150119

12. Fixed and bleached samples are blocked with 5% goat serum in PBSTw (0.3% - 0.5% Tween) for 1-2 hours.

13. Incubate the samples overnight with primary antibody in blocking solution (5% goat serum in PBSTw (0.3% - 0.5% Tween).

14. Wash the samples six times in PBSTw (0.3% - 0.5% Tween) for 20 minutes each.

15. Incubate overnight with appropriate secondary antibody (1:500 -1:1000) in blocking solution.

Note: DAPI (0.2 ug/mL – 1 ug/mL) can be added at this step.

16. Wash the samples six times in PBSTw (0.3% - 0.5% Tween) for 20 minutes each.

17. Optional: Post-fix the samples in 4% formaldehyde in PBSTx (0.3% Triton) for 20 – 30 minutes.

18. Clear the samples for 1-2 days in 20% Scale A2 + DABCO.

    Note: We have not observed any significance difference in immunostaining when PBSTx (0.3% - 0.5% Triton) is used in place of PBSTw (0.3% - 0.5% Tween).
