# Supplementary material for: A powerful and versatile new fixation protocol for immunostaining and in situ hybridization that preserves delicate tissues
Source: BMC Biol. 2024 Nov 4;22:252. doi: 10.1186/s12915-024-02052-3 (PMC11533299; doi:10.1186/s12915-024-02052-3)
Supplement: Supplementary file 16 — Additional file 16: Details of solutions used for fixation, WISH, and immunofluorescence. [file 12915_2024_2052_MOESM16_ESM.pdf]

<u>Solutions</u>

10X PBS

Reagent	Final concentration	Add to 1 L
NaCl	1.37 M	80 g
KCl	27 mM	2 g
KH2PO4	20 mM	2.4 g
Na2HPO4	100 mM	14.4 g
Milli-Q water		Fill to 1 L

Adjust pH to 7.4, filter, and store at RT.

PBSTw 0.3% / 0.5%

Reagent	Final concentration	Add to 1 L
10X PBS (filtered)	1X	100 mL
10% Tween 20 (filtered, protect from light)	0.3% or 0.5%	30 mL (for 0.3%) or 50 mL (for 0.5%)
Milli-Q water		870 mL (for 0.3%) or 850 mL (for 0.5%)

Store at RT.

PBSTx 0.3%/0.5%

Reagent	Final concentration	Add to 1 L
10X PBS (filtered)	1X	100 mL
10% Triton X 100	0.3% or 0.5%	30 mL (for 0.3%) or 50 mL (for 0.5%)
Milli-Q water		870 mL (for 0.3%) or 850 mL (for 0.5%)

Store at RT.

NA Solution

Reagent	Final concentration	Add to 10 mL
1 M HEPES-KOH, pH 7.5	100 mM	1 mL
16% PFA	4%	2.5 mL
0.5 M EGTA, pH 8.0	25 mM	0.5 mL
1 M MgSO4	50 mM	0.5 mL
70% HNO3	0.53%	0.075 mL
Milli-Q water		5.425 mL

Prepare fresh.

FA Solution

Reagent	Final concentration	Add to 10 mL
1M HEPES-KOH, pH 7.5	100 mM	1 mL
16% PFA	4%	2.5 mL
0.5 M EGTA, pH 8.0	25 mM	0.5 mL
96% Formic acid	4.80%	0.5 mL
Milli-Q water		5.5 mL

Prepare fresh.

Formamide bleach

Reagent	Final concentration	Add to 10 mL
30% H2O2	6%	2 mL
100% Formamide	1%	100 µL
PBSTx (0.5%)		8 mL

Prepare fresh.

Pre-Hybridization buffer (Pre-Hybe)

Reagent	Final concentration	Add to 500 mL
Formamide	50%	250 mL
10% Tween-20	1%	50 mL
20 X SSC	5X	125 mL
50 X Denhardts	1X	10 mL
Heparin	100 µg/mL	0.05 g
DTT	50 mM	3.85 g
Torula Yeast RNA	1 mg/mL	0.5 g
Milli-Q water		Up to 500 mL

Store at -20 °C.

Hybridization buffer (Hybe)

Reagent	Final concentration	Add to 500 mL
Formamide	50%	250 mL
10% Tween-20	1%	50 mL
20 X SSC	5X	125 mL
50 X Denhardts	1X	10 mL
Heparin	100 µg/mL	0.05 g
DTT	50 mM	3.85 g
Torula Yeast RNA	1 mg/mL	0.5 g
Dextran Sulfate	5%	25 g
Milli-Q water		Up to 500 mL

Store at -20°C.

Wash Hybe - 0.5% Tween-20

Reagent	Final concentration	Add to 500 mL
Formamide	50%	250 mL
10% Tween-20	0.50%	25 mL
20 X SSC	5X	125 mL
50 X Denhardts	1X	10 mL
Milli-Q water		Up to 500 mL

Store at -20°C.

0.2X SSC and 2X SSC + 0.1% Tween-20

Reagent	Final concentration	Add to 1 L
20X SSC	2X or 0.2X	100 mL (for 2X) and 10 mL (for 0.2X)
10% Tween-20	0.10%	10 mL
Milli-Q water		890 mL (for 2X) and 980 mL (for 0.2X)

Store at RT.

MABT

Reagent	Final concentration	Add to 1 L
Maleic acid	100 mM	11.61 g
2.5 M NaCl	150 mM	60 mL
10% Tween-20	0.10%	10 mL
Milli-Q water		Fill to 1 L

Adjust pH to 7.5 with NaOH and store at RT.

5% Horse serum + 0.5% RWBR Block

Reagent	Final concentration	Add to 500 mL
Horse serum (filtered)	10%	2.5 mL
10% Roche Western Blocking Reagent	0.50%	2.5 mL
MABT		45 mL

Make fresh.

Borate Buffer

Reagent	Final concentration	Add to 500 mL
NaCl	2 M	58.44 g
Boric Acid	0.1 M	3.09 g
Milli-Q water		Fill to 500 mL

Adjust pH to 8.5, filter, and store at 4 °C.

10% PVA

Reagent	Final concentration	Add to 500 mL
PVA	10%	50 g
Milli-Q water		Fill to 500 mL

Filter and store at RT.

NBT/BCIP Development Solutions

Reagent	Alkaline phosphatse (AP) Buffer	Equilibration (EQ) Buffer	Development (DEV) Buffer	Final concent ration
1 M Tris, pH 9.5 (filtered)	1.5 mL	1.5 mL	1.5 mL	0.1 M
2.5 M NaCl (filtered)	600 µL	600 µL	600 µL	0.1 M
1 M MgCl2 (filtered)	750 µL	750 µL	750 µL	0.05 M
10% Tween-20	150 µL	150 µL	150 µL	0.10%
Milli-Q water	12.3 mL	4.8 mL	NONE	
10% PVA	NONE	7.5 mL	12.3 mL	
BCIP	NONE	NONE	80 µL	
NBT	NONE	NONE	40 µL	
Total volume	15 mL	15 mL	15 mL	

Make fresh.

80% glycerol

Reagent	Final concentration	Add to 400 mL
100% glycerol	80%	320 mL
500 mM EDTA	0.8 mM	800 µL
1 M Tris pH 7.4	10 mM	4 mL
Milli-Q water		75.2 mL

Store at RT.

75% Scale A2

Reagent	Final concentration	Add to 300 mL
8M Urea	2 M	75 mL
100% Glycerol	75%	225 mL

Store at RT.

20% Scale A2 + DABCO

Reagent	Final concentration	Add to 500 mL
8M Urea	4 M	250 mL
10% TritonX	0.10%	5 mL
100% Glycerol	20%	100 mL
DABCO (antifade reagent)	2.50%	12.5 g
Milli-Q water		132.5 mL

Store at 4 °C

Rompolas relaxant solution

Reagent	Final concentration	Add to 10 mL
70% Nitric Acid	1%	140 µL
16% PFA	4%	532 µL
1 M MgSO4	50 mM	500 µL
1X Montjuic water		8.828 mL

Make fresh.

10X DNP/Fluorescein labeling mix

Reagent	Final concentration	Add to 71 µL
10 mM DNP-11-UTP / Fluorescein-12-UTP	3.5 mM	25.0 µL
100 mM UTP	6.5 mM	4.7 µL
100 mM ATP	10 mM	7.1 µL
100 mM GTP	10 mM	7.1 µL
100 mM CTP	10 mM	7.1 µL
ddH2O		20 µL

Store at -20 °C.
